# Evidence of secondary Notch signaling within the rat small intestine

**DOI:** 10.1242/dev.204277

**Published:** 2025-06-03

**Authors:** Eleanor Zagoren, Nicolas Dias, Anderson K. Santos, Zachary D. Smith, Nadia A. Ameen, Kaelyn Sumigray

**Affiliations:** ^1^ Department of Genetics, Yale School of Medicine, New Haven, CT 06510, USA; ^2^ Yale Stem Cell Center, Yale School of Medicine, New Haven, CT 06510, USA; ^3^ Department of Pediatrics/Gastroenterology and Hepatology, Yale School of Medicine, New Haven, CT 06510, USA; ^4^ Department of Cellular and Molecular Physiology, Yale School of Medicine, New Haven, CT 06510, USA

**Keywords:** Small intestine, Secretory cells, CFTR high expresser cells (CHEs), Notch signaling, Single-cell RNA-seq

## Abstract

The small intestine is well known for its nutrient-absorbing enterocytes; yet equally crucial for homeostasis is a diverse set of secretory cells, all presumed to originate from the same intestinal stem cell. Despite their major roles in intestinal function and health, understanding how the full spectrum of secretory cell types arises remains a longstanding challenge, largely due to their comparative rarity. Here, we investigate the specification of a rare population of small intestinal epithelial cells found in rats and humans but not mice: CFTR high expressers (CHEs). Using pseudotime trajectory analysis of single-cell RNA-sequencing data from rat jejunum, we provide evidence that CHEs are specified along the secretory lineage and appear to employ a second wave of Notch-based signaling to distinguish themselves from other secretory cells. We validate the transcription factors directing these cells from crypt progenitors and demonstrate that Notch signaling is necessary to induce CHE fate *in vivo* and *in vitro*. Our findings suggest that Notch reactivation along the secretory lineage specifies CHEs, which may help regulate luminal pH and have direct relevance in cystic fibrosis pathophysiology.

## Teaser

**Summary:** Rare CFTR high expresser cells, found in rats and humans but not mice, arise from the intestinal secretory lineage yet require active Notch signaling for fate specification.

## INTRODUCTION

The physiological function of an epithelial tissue directly depends on the frequency and spatial distribution of its differentiated cell types, properties that are meticulously maintained by dedicated stem cell populations. In the mammalian small intestine, the epithelium is completely renewed every 3-7 days throughout adulthood (Barker et al., 2007), experiencing substantial cellular turnover without compromising its tissue morphology or function. Specifically, stem cells localized to crypt compartments continuously give rise to terminally differentiated cells that occupy the villus.

The primary role of the small intestine is to absorb nutrients from digested food. The function of secretion, however, is equally important for homeostasis, as it allows the intestinal epithelium to sense its highly dynamic luminal environment and release various ions, fluids and molecules to modify its surroundings precisely. These key functions are facilitated by specialized cell types. Intestinal epithelial differentiation is tightly regulated along two distinct cell fate lineages: absorptive and secretory. While absorptive cells are exclusively enterocytes, secretory cell types are varied and include anti-microbial Paneth cells, mucus-secreting goblet cells, hormone-secreting enteroendocrine cells (EECs) and chemosensory tuft cells (Gehart and Clevers, 2019).

The diverse secretory lineage is thought to arise from a single secretory progenitor (Yang et al., 2001; Yan et al., 2012). The fundamental determinant of whether a cell will become an absorptive or secretory progenitor is Notch signaling via lateral inhibition, with absorptive progenitors ‘Notch-on’ and secretory progenitors ‘Notch-off’ (Yang et al., 2001; Fre et al., 2005). In the absorptive progenitor, Notch receptors regulate cell fate by promoting expression of the transcription factor Hes1, which initiates an extensive transcriptional program upon Notch activation. Conversely, in the secretory progenitor, Notch ligands induce a Notch-off state, promoting the expression of the transcription factor Atoh1 to establish a secretory identity (Lo et al., 2017). Atoh1 has been described to be the master regulator of the secretory lineage, and it and Hes1 demonstrate reciprocal repression to reinforce cell fate decisions along their respective lineages (Yang et al., 2001). Loss of Atoh1 has been described to result in decreased populations of goblet cells, Paneth cells and EECs (Yang et al., 2001), although the latter group is exceptionally diverse in terms of cell subtypes (Haber et al., 2017) and has also been described to be specified in an Atoh1-independent manner (Gracz et al., 2018). Finally, the rare chemosensory population of tuft cells remains one of the least well understood intestinal secretory cell types in terms of lineage dynamics, although they are thought to be specified independently of Atoh1 (Bjerknes et al., 2012; Gracz et al., 2018) and other major secretory transcription factors.

A less-appreciated intestinal cell type with a putative secretory role and yet to be integrated into existing models of intestinal cell fate specification is the Best4^+^/CFTR high expresser (CHE). While present in humans (Busslinger et al., 2021; Strong et al., 1994; Burclaff et al., 2022), rats (Ameen et al., 1995), fish (Hiroi et al., 2005; Sur et al., 2023) and other vertebrates, an analogous cell type has not been found within the mouse intestine. As much of our knowledge of cell fate specification in the intestine has been informed by lineage tracing in genetic mouse models, we have limited understanding of the mechanisms guiding CHE specification. Furthermore, the lack of tools to perturb CHE fate specification has precluded our ability to enrich for these cells and test their physiological contribution within the intestine. Although high expression of the chloride and bicarbonate ion channel CFTR (cystic fibrosis transmembrane conductance regulator; Ameen et al., 1995) in CHEs hints at the capacity for intense secretory activity and a role in pH regulation, the function of CHEs and the mechanisms by which they are specified remain unknown.

In this study, we aim to explore CHE fate specification and integrate CHEs into the broader hierarchy of secretory cells. The rat intestine is an ideal system to study this, as the rat intestine (1) specifies CHEs, unlike the mouse intestine; (2) provides easily accessible tissue; and (3) has previously optimized CFTR antibodies (Golin-Bisello et al., 2005). Furthermore, we have developed a novel rat intestinal organoid system that allows for interrogation of CHE cell fate (Zagoren et al., 2023). Specifically, we use pseudotime analysis of single-cell RNA-sequencing data from rat intestinal jejunum to provide evidence that CHEs are specified along the secretory lineage. We further validate a set of distinguishing transcription factors and additional proteins to describe the biology of these cells from their initial induction within the crypt to their maturation within the villus. Unexpectedly, we identify an unusual reactivation of specific Notch receptors and effector proteins that strongly suggest the repurposing of this pathway to support CHE fate from a putative CHE-tuft progenitor state. Together, our work reveals that active Notch signaling is necessary to induce CHE fate. This paradoxical reactivation of Notch signaling within the secretory lineage broadens our appreciation for the dynamic regulatory processes that diversify complex tissues.

## RESULTS

### Rat CFTR CHEs are derived from the secretory lineage

**Fig. 1.**
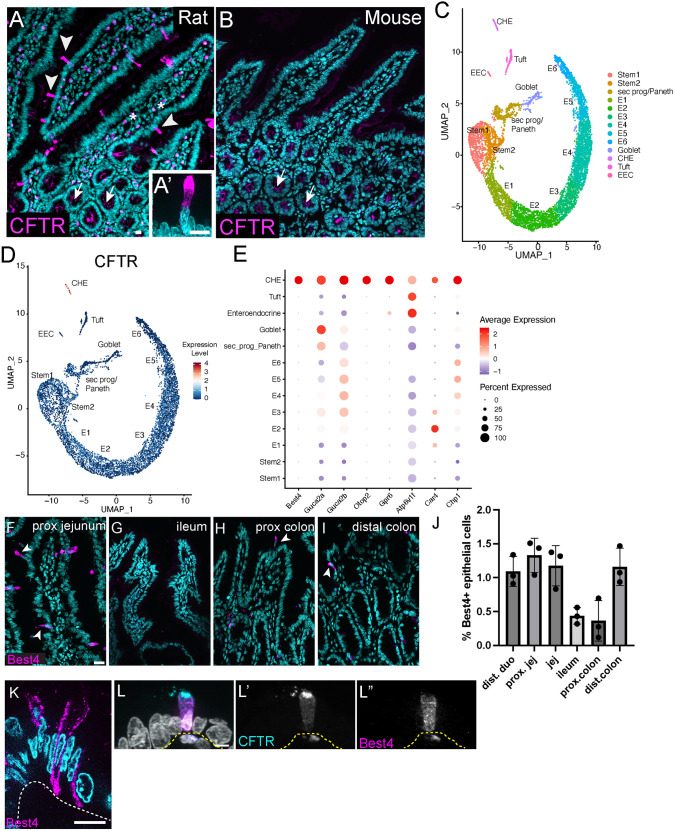
**CHEs are a transcriptionally distinct intestinal epithelial cell type in rats but not mice.** (A) Rat intestinal jejunum stained for CFTR (magenta). Arrowheads denote CHEs in differentiated villi. Arrows indicates CFTR enriched in crypts. Asterisks denote mesenchymal autofluorescence from immune cells. DAPI in cyan. (A′) CHE at higher magnification. (B) Mouse intestinal jejunum lacks CHEs when stained for CFTR (magenta). Arrows indicate CFTR enriched in crypts. (C) UMAP of rat intestinal jejunum epithelial cells (see Fig. S1A-H for clustering annotation). E1-E6 denote enterocytes at increasing stages of maturation. sec prog, secretory progenitor. (D) UMAP color-coded for CFTR expression across intestinal epithelial cell types. (E) Dot plot from scRNA-seq data demonstrating enrichment for proton-sensitive genes associated with pH regulation and ion and fluid homeostasis across epithelial cell types. (F-I) Bestrophin 4^+^ cells (Best4, magenta) in rat proximal jejunum (F), rat ileum (G), rat proximal colon (H) and rat distal colon (I). DAPI in cyan. (J) Percentage of Best4^+^ epithelial cells for each gut region. *n*=3 animals per region; >10,000 epithelial cells counted per animal per region. (K) High-magnification image of a single *z*-slice of a Best4^+^ cell. Best4 in magenta, DAPI in cyan. Note basolateral Best4 localization. Dashed line delineates basement membrane. (L-L″) Co-staining of rat proximal jejunum for CFTR (cyan in L, gray in L′) and Best4 (magenta in L, gray in L″). DAPI in gray in L. Scale bars: 10 μm.

CHEs have been previously characterized as regionally restricted, CFTR^+^ cells within rat and human duodenum and proximal jejunum, but they are strikingly absent from the mouse intestine (Jakab et al., 2013; Busslinger et al., 2021; Ameen et al., 2000, 1995; Strong et al., 1994). We validated the presence of the CHE population in the rat *in vivo*, where CFTR was highly enriched in CHEs in upper crypts and differentiated villi (Fig. 1A). In contrast, CFTR expression in mouse intestine was highest within crypts and apparent at low levels in the villus brush border, but not within discrete cell populations (Fig. 1B). Although CHEs have been hypothesized to regulate fluid secretion and/or luminal pH, their functional contributions to intestinal physiology are poorly understood, in part because we lack understanding of how they are specified.

To characterize CHE gene expression more deeply, we performed unbiased transcriptomic analysis with droplet-based 3′ single-cell RNA sequencing (scRNA-seq) on 7362 jejunal epithelial cells from two Sprague-Dawley rats. Clusters were first analyzed for expression of epithelial (Epcam), immune and blood markers (Fig. S1), and clusters expressing immune and blood markers that were negative for Epcam were removed. Remaining epithelial clusters were annotated using known marker gene expression as a means of distinguishing distinct states (Fig. 1C, Fig. S2A-H). A cluster of 88 cells were first defined as CHEs by their high *Cftr* expression (Fig. 1D). This is lower than the frequency of CHE cells described *in vivo* (Jakab et al., 2013), yet other studies have demonstrated that ‘fragile’ cells, such as pulmonary ionocytes, which are morphometrically similar to CHEs, both having long protrusions, are less well-represented in scRNA-seq experiments (Yuan et al., 2023). We confirmed that the CHE cluster did not express markers of other differentiated cell types or enterocyte-associated genes, as previously described by immunofluorescence (Ameen et al., 1995) (Fig. S2I,J). Instead, CHEs notably expressed multiple proton-sensitive genes implicated in pH regulation and ion and fluid homeostasis, including the bicarbonate transporter *Best4*, peptide hormones *Guca2a* (encodes guanylin) and *Guca2b* (encodes uroguanylin), and the ion channel *Otop2* (otopetrin 2) (Fig. 1E).

These data are consistent with rat CHEs being analogous to the BEST4^+^ ‘BCHE’ population recently characterized in the human proximal small intestine (Busslinger et al., 2021; Burclaff et al., 2022). We confirmed that Best4^+^ cells were most abundant in the proximal small intestine, consistent with CHE localization (Jakab et al., 2013), as well as the distal colon (Fig. 1F-J). High-magnification images of single slices of Best4^+^ cells in proximal jejunum demonstrate its basolateral membrane localization (Fig. 1K). Co-labeling of Best4 and CFTR in rat proximal small intestine confirmed that Best4^+^ cells in the small intestine and CHEs are indeed the same cell type (Fig. 1L), with CFTR enriched in the apical and subapical domains (Fig. 1L′) and Best4 enriched in the basolateral domains (Fig. 1L″). Taken together, CHEs broadly resemble a highly secretory cell type, with specific trafficking of directional ion channels to modulate the intestinal lumen.

### CHEs have a distinct differentiation trajectory among secretory cells

**Fig. 2.**
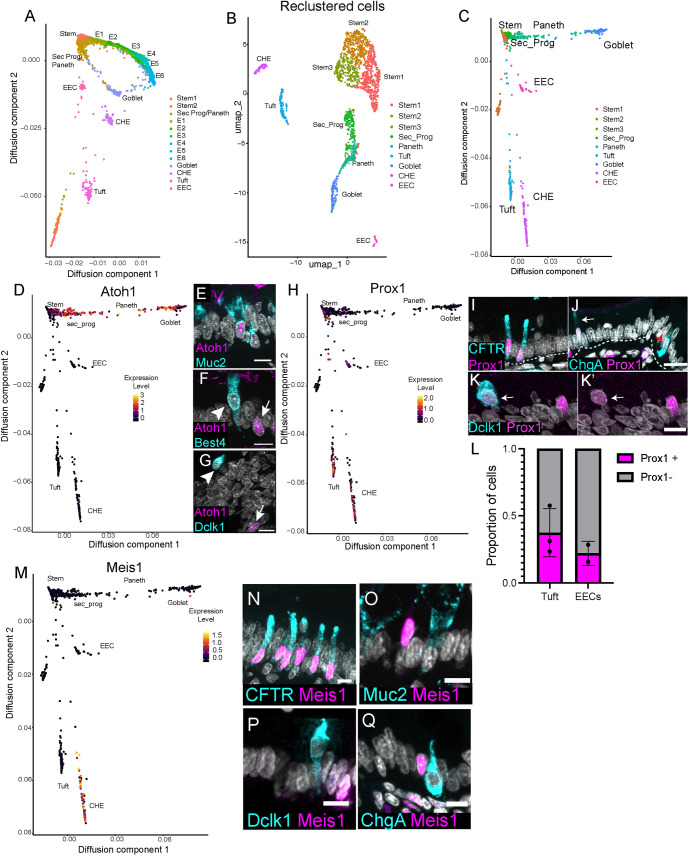
**CHEs derive from a distinct branch of the secretory lineage shared with tuft cells and EECs and are enriched for the transcription factors Meis1 and Prox1.** (A) Diffusion maps of rat intestinal epithelial cells, showing their differentiation as a measure of pseudotime. E1-E6 denote enterocytes of increasing maturation. (B) Reclustered UMAP of cells arising from stem and secretory progenitor clusters. (C) Diffusion maps of stem cells and secretory progenitors as a readout of pseudotime. Note the clear bifurcation between goblet/Paneth and EEC/tuft/CHE lineages. (D) Diffusion map color-coded for Atoh1 expression. (E-G) Atoh1 (magenta) staining for Muc2 (cyan, goblet cells; E), Best4 (cyan, CHEs; F) and Dclk1 (cyan, tuft cells; G). Arrows and arrowheads indicate Atoh1^+^ cells and cell type of interest, respectively. (H) Diffusion map of stem and secretory cells color-coded for Prox1 expression. (I) CFTR (cyan, CHE marker) and Prox1 (magenta) co-staining. (J) ChgA (cyan, EEC marker) and Prox1 (magenta) co-staining. Arrow indicates a Prox1^+^/ChgA^+^ cell, red arrowhead marks a Prox1^−^/ChgA^+^ cell. (K-K′) Dclk1 (cyan, tuft cell marker) and Prox1 co-staining (magenta; K′) (arrow)*.* (L) Proportion of tuft cells and EECs in rat proximal jejunum that are Prox1^+^. *n*=3 animals for tuft cells; *n*=2 for EECs. (M) Diffusion map of stem and secretory cells color-coded for Meis1 expression. (N) Meis1 (magenta) and CFTR (cyan, CHE marker) co-staining. DAPI in white. (O-Q) Meis1 (magenta) staining with Muc2 (cyan, goblet cells; O), Dclk1 (cyan, tuft cells; P) or ChgA (cyan, EECs; Q). Scale bars: 10 μm.

Rat CHEs are functionally characterized as secretory cells (Jakab et al., 2013), and previous studies in human intestine suggest that small intestinal CHEs arise from secretory progenitors (Burclaff et al., 2022). Although outside the scope of this study, colon BEST4^+^ cells with otherwise distinct transcriptional features are derived from the absorptive lineage in the human colon (Parikh et al., 2019). In our data set, we observed a clear disparity between a densely sampled, continuous enterocyte trajectory and multiple disconnected clusters of differentiated secretory cells. Paneth and goblet cells were obviously connected through a proliferative secretory progenitor. However, tuft cells, EECs and CHEs showed no obvious connectivity to each other or to a distinct progenitor or stem population (Fig. 1C, Fig. S2J), making it unclear whether CHEs derive from the secretory lineage or the absorptive lineage. Based on data from the human small intestine (Burclaff et al., 2022), we hypothesized that CHEs arise from the secretory lineage. To test this, we generated diffusion maps of rat intestinal epithelial cells as a measure of pseudotime (Fig. 2A). Diffusion component 1 (DC1) largely captures differentiation of the absorptive lineage, while Diffusion component 2 (DC2) distinctly separates major secretory cell types, including CHEs.

To examine more sensitively the transcriptional relationships between distinct secretory cell types, we first subsetted stem and secretory populations and reclustered the data (Fig. 2B, Fig. S3A-G). With the overpowering enterocyte populations removed, we were able to segregate a small secretory progenitor population, detectable by Dll1 expression (van Es et al., 2012) and cell cycle stage (Fig. S3H,J). Unlike the differentiated cells of the intestine, which were post-mitotic in G0, the secretory progenitor cluster contained cycling cells in both S and G2/M (Fig. S3J). *In vivo*, secretory progenitors have been described as mostly restricted to the crypt region above the uppermost Paneth cell and are marked by Dll1 expression (Sangiorgi and Capecchi, 2008; Yan et al., 2012; van Es et al., 2012). We also observed this population of secretory progenitors in the rat, which expressed high levels of Dll1 protein (Fig. S3K). Furthermore, these Dll1^+^ secretory progenitors were actively cycling, as evidenced by Ki67 (*Mki67*) expression (Fig. S3L).

We performed pseudotime analysis of the stem cells and secretory cells by generating diffusion maps to identify clear subtrajectories in their respective transcriptional behaviors. Interestingly, our diffusion map demonstrated a clear bifurcation between the goblet/Paneth and the CHE/tuft/EEC lineages (Fig. 2C), suggesting either the presence of multiple discrete progenitor cell types or a stepwise partitioning of a multipotent progenitor into more specialized states. To identify genes contributing to this bifurcation in downstream secretory cell types, we compared gene expression between the two groups, focusing particularly on transcription factors. As expected, the most highly enriched transcription factor along the goblet/Paneth lineages was Atoh1 (Fig. 2D), a factor with a well-known role in consolidating a Notch-off state to support secretory cell differentiation (Yang et al., 2001). Immunofluorescence confirmed that Atoh1 protein was expressed in goblet cells (Fig. 2E) but was absent from CHEs and Tuft cells (Fig. 2F,G). In contrast, the transcription factor Prox1 was the most highly enriched gene shared across the CHE/tuft/EEC branch (Fig. 2H), suggesting two distinct secretory axes. Overall, our results reveal an intriguing bifurcation in transcriptional regulatory networks distinguishing CHE/tuft/EECs from canonically Atoh1^+^ goblet/Paneth cells.

The restricted expression of Prox1 to CHEs, tuft cells and EECs was particularly interesting, as the fly homolog of Prox1, Prospero, plays an important role in EEC fate in *Drosophila* adult midgut (Zeng and Hou, 2015). Prox1 has also been previously shown to mark some tuft cells and EECs in mouse (Yan et al., 2017). Immunofluorescence of rat proximal jejunum further confirmed the expression of Prox1 in CHEs (Fig. 2I) and some EECs and tuft cells (Fig. 2J-L).

The shared expression of Prox1 within a distinct sub-branch of the secretory lineage prompted us to search for additional factors that might support cell fates downstream of Prox1 induction. To understand how CHE fate is uniquely specified, we performed differential gene expression analysis of the CHE cluster compared to other cell states and manually curated a list of candidate transcription factors as likely drivers of cellular identity. Notably, *Meis1* was the sole transcription factor candidate exclusive to CHEs and not found in any other intestinal epithelial cell type (Fig. 2M, Fig. S3M). Immunofluorescence confirmed that Meis1 protein is specifically expressed in CHEs, marked by CFTR (Fig. 2N), and not in other secretory cell types, including goblet cells (Fig. 2O), tuft cells (Fig. 2P) and EECs (Fig. 2Q). We also examined Best4^+^ colon cells. While they have some overlapping gene expression with small intestinal CHEs (namely Best4 and Otop2), they do not express CFTR-like small intestinal CHE/BCHEs (Burclaff et al., 2022). Surprisingly, while Best4^+^ colon cells were Prox1^+^ (Fig. S3N), Meis1 protein was undetectable (Fig. S3O), even though previous transcriptomic analysis had identified it as an enriched gene (Parikh et al., 2019). These results identify Meis1 as a small intestinal CHE marker that may play an important role in CHE differentiation and functional identity.

### CHEs express markers of active Notch signaling

Atoh1 is expressed in canonical secretory cells in the intestine, where it reinforces the inhibition of Notch signaling and its associated transcriptional program (Yang et al., 2001; Lo et al., 2017). In contrast, Atoh1 expression is repressed in the absorptive lineage by the downstream Notch target Hes1 (Yang et al., 2001), making Notch signaling a well-established driver of the decision to become an absorptive or secretory progenitor (Yang et al., 2001; Gehart and Clevers, 2019). However, our data above demonstrate that the CHE/tuft/EEC branch of the secretory lineage does not express Atoh1 (Fig. 2D-G), raising uncertainty about the role of Notch repression in maintaining cell fates for this branch of the secretory lineage. Therefore, we aimed to determine the Notch signaling status of cells within the CHE/tuft/EEC branch.

**Fig. 3.**
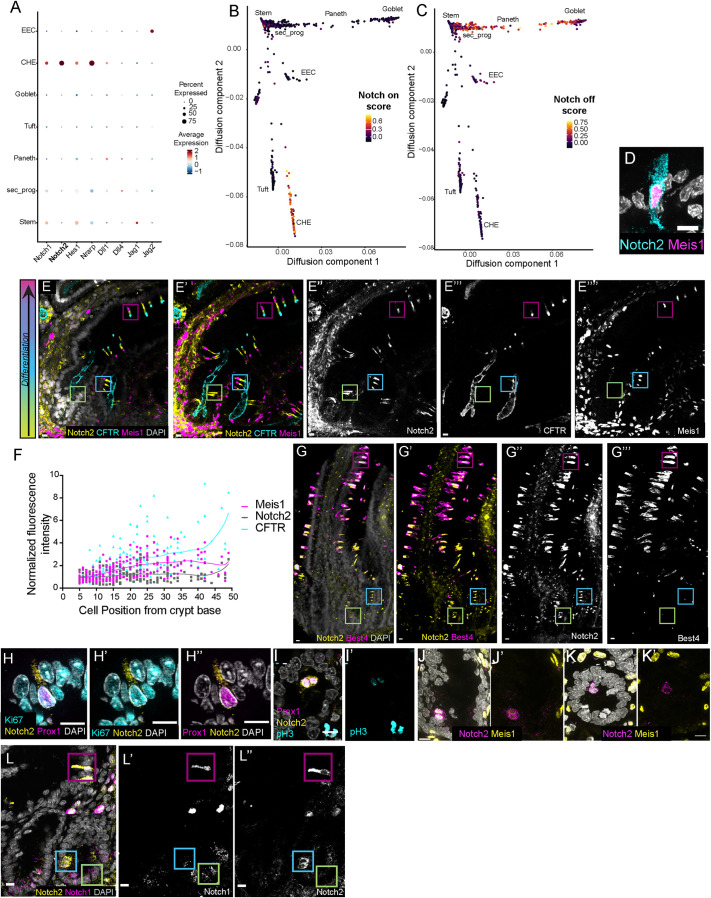
**Notch2 is a candidate marker of CHE progenitors.** (A) Dot plot from scRNA-seq data showing relative expression of factors associated with Notch signaling across intestinal epithelial cell types. (B,C) Module scores for Notch-on (B) and Notch-off (C) gene signatures. (D) Meis1 (magenta, CHEs) and Notch2 receptor (cyan) co-staining. DAPI in white. (E-E⁗) 3D whole-mount rat proximal jejunum stained for Notch2 (yellow in E,E′; white in E″), CFTR (cyan in E,E′, white in E‴) and Meis1 (magenta in E,E′, white in E⁗). Color of box denotes approximate localization along the crypt-villus axis (left). DAPI in white. (F) Quantified fluorescence intensity for cells in each position along the crypt villus axis of 3D whole-mounts for Meis1 (magenta), CFTR (cyan) and Notch2 (black). Note the increase in fluorescence intensity with increasing position from the crypt base. Normalized to mean intensity of cell positions 5-7 for *n*=3 animals. (G-G‴) 3D whole-mount rat proximal jejunum stained for Notch2 (yellow in G,G′, white in G″) and Best4 (magenta in G,G′, white in G‴). DAPI in white. (H-H″) Notch2^+^/Prox1^+^ cell expressing Ki67. Notch2 in yellow, Prox1 in magenta, Ki67 in cyan. (I,I′) Notch2^+^/Prox1^+^ crypt cells in active mitosis. Note symmetric cell division for both Notch2 (yellow) and Prox1 (magenta). (I′) Note the low level of phospho-histone 3 (in cyan) as cell is exiting mitosis. (J,J′) Notch2^+^/Meis1^Low^ cells in active mitosis. Notch2 (magenta), Meis1 (yellow), DAPI (white). (K,K′) Notch2^+^ cell lacking Meis1 in active mitosis. Notch2 (magenta), Meis1 (yellow), DAPI (white). (L-L″) Actively dividing Notch2^+^ (yellow in L, white in L″) crypt cells are Notch1^−^ (magenta in L, white in L′), and distinct from crypt base stem cells, which only express Notch1. DAPI in white. Scale bars: 10 μm.

Unexpectedly for a cell type arising from the secretory lineage, scRNA-seq analysis revealed that CHEs expressed factors associated with active Notch signaling, including the *Notch1* and *Notch2* receptor genes (Fig. 3A, Fig. S4). More broadly, when we applied collective modules for Notch-on and Notch-off gene signatures as described in the Materials and Methods, we observed that the Notch-on signature was high within the CHE branch (Fig. 3B), while a clear Notch-off signature was apparent in the goblet/Paneth branch (Fig. 3C). Furthermore, at the transcriptomic level, our scRNA-seq analysis indicated that CHEs were the only cells in the rat intestine highly enriched for the *Notch2* receptor (Fig. 3A, Fig. S4H). Surprisingly, Notch2 was not strongly expressed in the stem cells of the rat intestine, whereas the Notch1 gene was expressed in both CHEs and stem cell populations (Fig. S4G). This absence of Notch2 in intestinal stem cells has also been described in human intestine (Tsai et al., 2022), but is contrary to reports in mouse intestine (Riccio et al., 2008; Carulli et al., 2015) where Notch1 and Notch2 are co-expressed in intestinal stem cells and function redundantly. We confirmed by immunofluorescence that CHEs specifically expressed the Notch2 receptor protein (Fig. 3D). Notch2 has been historically shown to specify cell types independently of the Notch1 receptor (Jeliazkova et al., 2013; Basil et al., 2022; Nakano et al., 2022), and we were intrigued by the possibility of a Notch2-specific role in CHE fate specification.

### Notch2 is a candidate marker of CHE precursors

Intestinal epithelial cells progressively differentiate as they migrate upwards from the stem cell/progenitor zone in the crypt towards villi. In the absence of straightforward genetic lineage-tracing tools in the rat, we took advantage of the position of cells along this progressively differentiating crypt-villus axis to infer the general spatial and temporal properties of CHE differentiation. Using 3D whole-mounts of rat jejunum, which preserve entire crypt-villus axes, we stained for CFTR and our identified CHE markers to determine the dynamics of CHE protein expression along the CHE differentiation axis. We first examined CFTR, Notch2 and Meis1 (Fig. 3E-E⁗) and observed that, as expected, CHEs in the differentiated villus region expressed high levels of all three proteins (Fig. 3E-E⁗, magenta box, F). Similarly, labeling for Best4 and Notch2 (Fig. 3G-G‴) clearly identified double-positive CHEs within the villus (Fig. 3G-G‴, magenta box).

Remarkably and unexpectedly, we observed an additional population of Notch2^+^ cells in the proliferative crypt region (Fig. 3E,E′, green box) that did not express CFTR (Fig. 3E‴) or Meis1 (Fig. 3E⁗). These Notch2^+^ cells localized within the mid and upper crypts and progressively expressed increasing levels of CFTR and Meis1 as they ascended the crypt-villus axis (Fig. 3F). Notably, the lowest crypt position occupied by these Notch2^+^ cells was the +5 position, which is immediately proximal to the Dll1^+^ secretory progenitor at the +4 position (Fig. S3K). Best4 expression was also restricted to the villus compartment (Fig. 3G,G′,G‴), consistent with computational data from the human intestine suggesting that BEST4 is a mature CHE marker (Burclaff et al., 2022).

Interestingly, a Prox1^+^ progenitor has been previously described in the mouse with both reserve stem cell activity and rare but detectable clonogenic potential during homeostasis (Yan et al., 2017). We found that the Notch2^+^ crypt cells were positive for Prox1 (Fig. 3H,I). This raises the intriguing hypothesis that the Notch2^+^/Prox1^+^/Best4^−^/CFTR^−^ cells we observed in the crypt region may function as a candidate progenitor, although it is also possible that these cells are simply immature CHEs that have not reached full differentiation status.

To examine whether Notch2^+^/Prox1^+^ crypt cells in the rat could act as a progenitor population, we first aimed to determine whether these cells were proliferative. We co-stained intestines for Notch2, Prox1 and Ki67 and found that a subset of Notch2^+^/Prox1^+^ cells was Ki67^+^ (Fig. 3H-H″), suggesting that this population was still in the cell cycle and had not terminally differentiated and entered G0. In contrast, Ki67^+^ cells were not observed in differentiated villar CHEs (Fig. S3I,I′), and our scRNA-seq data indicated that differentiated CHEs fully exist within the G0/G1 stage of the cell cycle (Fig. S3J). Although rare, we also detected Notch2^+^/Prox1^+^ crypt cells in active mitosis (Fig. 3I,I′). In these cases, Notch2 and Prox1 were expressed in both daughter cells, suggesting that Notch2^+^/Prox1^+^ cells undergo symmetric divisions. Next, we examined Meis1 levels in mitotic cells, and found that some Notch2^+^ cell pairs were Meis1^+^ (Fig. 3J,J′), while others were Meis1^−^ (Fig. 3K,K′). We did not observe any instances in which dividing cells were asymmetrically Meis1^+^. Co-staining for Notch1 and Notch2 revealed that actively dividing Notch2^+^ crypt cells were Notch1^−^ (Fig. 3L-L″, blue box) and distinct from stem cells in the crypt base, which only expressed the Notch1 receptor (Fig. 3L-L″, green box). These data suggest that Notch2^+^ cells are proliferative and arise within the crypt, and that, rather than simply being immature CHEs, they may maintain the potential to generate other cell types within the CHE/tuft/EEC axis. The distinct expression patterns of Notch1 and Notch2 also suggest that Notch2^+^ CHE progenitors do not simply maintain active Notch signaling but reactivate it through Notch2 expression.

### CHE fate specification requires active Notch signaling

**Fig. 4.**
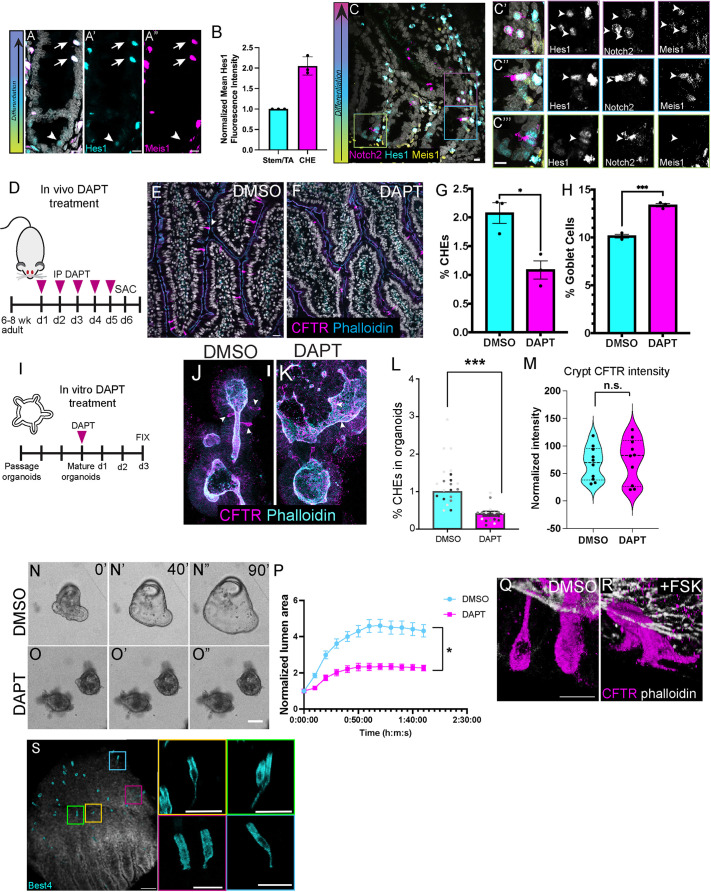
**CHE specification requires active Notch signaling.** (A-A″) Meis1 (magenta, CHE marker; arrowheads) and Hes1 (cyan) co-staining. Stem/transit amplifying (TA) cells with positive Hes1 staining are marked by arrowheads. Scale bars: 10 μm. (B) Normalized mean fluorescence intensity of nuclear Hes1 in CHEs and stem/TA cells. *n*=3 animals. (C-C‴) Nuclear Hes1 (cyan) is present in crypt cells positive for Notch2 (magenta), which have not yet upregulated Meis1 (yellow). DAPI in white. Color of box denotes approximate localization along the crypt-villus axis (left). (D) Schematic of *in vivo* DAPT treatment. d, day; IP, intraperitoneal; SAC, sacrificed. (E,F) Immunofluorescence staining for CHEs (magenta, CFTR) and F-actin (cyan, phalloidin) for vehicle (E) and DAPT-treated (F) rats. DAPI in white. Scale bar: 10 μm. (G,H) Percentage of CHEs (G; *P<0.01) and percentage of goblet cells (H; ***P<0.0003) in DAPT-treated rats relative to DMSO controls. *n*=3 rats per treatment. For each animal, images were acquired for at least eight different ROIs. (I) Schematic of *in vitro* DAPT treatment. (J,K) Immunofluorescence staining for CHEs (magenta, CFTR) for control (J) and DAPT-treated (K) rat intestinal organoids. Arrowheads mark CHEs. Scale bar: 20 µm. (L) Quantification of normalized CHE abundance in DMSO versus DAPT-treated organoids. *n*=3 replicates from two independently derived organoid lines. Each point denotes one organoid. ***P<0.0006. (M) Quantification of normalized crypt CFTR intensity for DMSO (cyan) and DAPT-treated (magenta) organoids. *n*=3 replicates; n.s., not significant. (N-O″) Forskolin swelling for control (N-N″) and DAPT-treated (O-O″) rat intestinal organoids. 0′, 0 min after DAPT treatment; 40′, 40 min post-treatment; 90′, 90 min post-treatment. Scale bar: 100 μm. (P) Normalized lumen area for DMSO (control) versus DAPT-treated organoids over time following forskolin swelling. *n*=3 replicates. For each replicate, at least 20 organoids were quantified. Unpaired *t*-test, area under the curve at 110 min; *P<0.01. (Q,R) CHE morphology in organoids with DMSO treatment (Q) and with forskolin (FSK) treatment (R). CFTR (magenta) marks CHEs, phalloidin (gray) marks the apical domain. Scale bar: 10 µm. (S) Best4^+^ CHEs (cyan) display long protrusions *in vivo*. 3D projections of individual cells with protrusions are shown at high magnification on the right. Scale bars: 25 µm.

While the presence of the Notch2 receptor strongly suggests active Notch signaling in CHEs, receptor expression does not necessarily reflect downstream Notch signaling activity. Therefore, we tested whether Hes1, the dominant effector transcription factor for active Notch signaling in the intestine, was expressed in CHEs. Co-staining of Hes1 and Meis1 revealed that CHEs express high levels of nuclear Hes1 (Fig. 4A, arrows), strongly implying that CHEs engage in active Notch signaling. We also confirmed nuclear Hes1 signal in proliferative Meis1^−^ crypt cells as a positive control (Fig. 4A′, arrowheads), as the requirement for Notch signaling in intestinal stem cell homeostasis and transit amplifying cells has been well-characterized (Pellegrinet et al., 2011). Interestingly, the fluorescence intensity of nuclear Hes1 in CHEs was significantly higher than that of stem/transit amplifying cells (Fig. 4B), supporting the idea that Notch signal transduction is an active part of CHE differentiation. Nuclear Hes1 was also present in the Notch2^+^ crypt cells (Fig. 4C), consistent with the notion of these cells as potential CHE progenitors in which Notch signaling is already active.

To test whether Notch signaling was necessary for CHE fate, we injected adult rats for five consecutive days with DAPT (Fig. 4D), a γ-secretase inhibitor that blocks the cleavage of the Notch intracellular domain, thereby inhibiting downstream Notch signaling. The experimental timeline accounts for the complete renewal of the intestinal epithelium every 3-7 days (Barker et al., 2007). DAPT treatment significantly decreased CHE number relative to the DMSO control, demonstrating that CHE fate specification requires active Notch signaling (Fig. 4E-G). Conversely, we found that goblet cell number increased upon Notch inhibition (Fig. 4H), as expected from previous reports (Yang et al., 2001) and consistent with the relative positioning of CHE versus goblet cells according to our diffusion component mapping.

To more robustly test mechanisms driving fate specification of CHEs and, ultimately, CHE function, we generated intestinal organoids from the rat jejunum (Zagoren et al., 2023). We first aimed to determine whether CHEs were specified in rat organoid models. Undifferentiated intestinal spheroids present 2 days after culturing did not contain CHEs (Fig. S5A,B). However, upon organoid budding and differentiation, CHEs were clearly defined (Fig. S5C,D). CHEs in organoids localized to the same domains as *in vivo*, including enrichment within the differentiated domains of the upper crypt and villus regions (Fig. S5E). Outside of CHEs, rat organoid enterocytes also expressed baseline CFTR in a gradient similar to that seen *in vivo*, with highest levels in the crypt and low levels in villar domains (Fig. S5F). CHEs in organoids also expressed Meis1, Prox1, Best4 and Notch2, as *in vivo* (Fig. S5G-J). We then verified whether Notch signaling regulates CHE fate *in vitro* as it does *in vivo*. We allowed organoids to differentiate, then treated them with 5 µM DAPT for 3 days (Fig. 4I). After DAPT treatment, we assessed the relative abundance of CHEs in DAPT versus vehicle (DMSO) treatment. As found *in vivo*, DAPT treatment significantly inhibited CHE specification (Fig. 4J-L). These data suggest that active Notch signaling is required for CHE fate specification in organoids, as it is within intact tissue. Outside of CHEs, most CFTR is expressed within the crypt domain. To confirm that DAPT treatment did not globally affect CFTR expression, particularly because Notch signaling plays important roles in stem cell maintenance, we measured the fluorescence intensity of CFTR within crypts of vehicle and DAPT-treated organoids. No significant difference was observed in normalized crypt CFTR intensity (Fig. 4M). Concurrently with these studies in rat intestinal organoids, a recent study demonstrated with DAPT treatment that Notch is necessary for CHE cell fate in human intestinal organoids (Wang et al., 2025).

Given the relative ease with which we could chemically modulate CHE number within organoids, we next sought to evaluate a potential secretory function for CHEs and their contribution to fluid secretion. Forskolin, an adenylyl cyclase activator, is commonly used in organoid-based assays to test for the ability to swell and secrete fluid (Boj et al., 2017). Vehicle (DMSO) or DAPT-treated organoids were treated with forskolin to induce fluid secretion into the lumen, and the degree of organoid swelling was observed. Notably, decreased CHE specification in DAPT-treated organoids resulted in strong abrogation of organoid swelling (Fig. 4N-P), indicating a dramatic change in flux between the external medium and the luminal space and supporting a role for CHEs in sustained fluid secretion in intestinal organoids. The similar intensities of CFTR staining in crypts between control and DAPT-treated organoids (Fig. 4M) leads us to suggest that the secretory defects observed in DAPT-treated organoids are not attributable to CFTR loss in the crypt compartment but rather to reduced CHE numbers. Surprisingly, when we stained rat organoids for CFTR to examine CHEs after forskolin activation, we found that they had dramatically changed shape, forming long protrusions (Fig. 4Q,R). In fixed tissue *in vivo*, we could occasionally capture CHEs with long protrusions (Fig. 4S), similar to what has been described for rare CFTR-enriched pulmonary ionocytes in the airway (Yuan et al., 2023) and suggesting that CHEs can sense and respond to their environment.

**Fig. 5.**
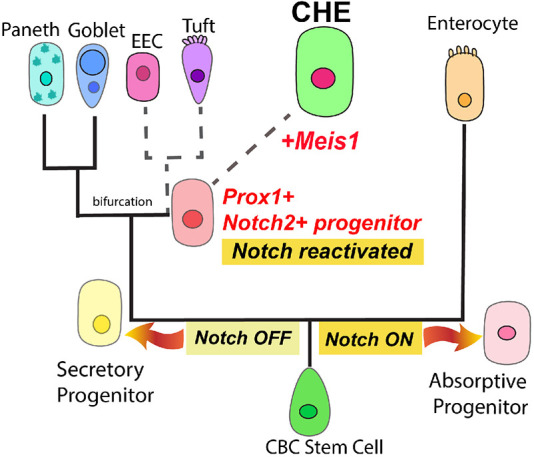
**Working model for CHE cell fate specification.** Working model of CHE cell differentiation in which: (1) CHEs arise from the secretory lineage, which is initially Notch off, (2) Notch is reactivated along the secretory lineage as a potentially multipotent Notch2^+^/Prox1^+^ progenitor and (3) Meis1 is upregulated to promote the expression of differentiated CHE markers, including CFTR and Best4. Dashed lines indicate proposed lineage relationships. CBC, crypt base columnar.

In summary, CHEs comprise a rare cell type in the proximal small intestine that likely arises from the secretory lineage along a distinct branch shared with tuft cells and EECs. CHEs are specifically enriched for Meis1 and share expression of Prox1 with tuft and EEC populations. Notch2 is a candidate marker of CHE progenitors, as we observe Notch2^+^/Prox1^+^ cells in crypts that lack enrichment for the CHE markers Best4 and CFTR. Finally, Notch signaling is necessary for CHE cell fate. These data support a model in which CHEs arise from the secretory lineage, yet, paradoxically, Notch is reactivated to specify this rare population (Fig. 5).

## DISCUSSION

In this study, we found that rare intestinal epithelial CHEs originate from the secretory lineage and depend on active Notch signaling for their fate specification. We used scRNA-seq from epithelial cells of rat jejunum and generated diffusion maps of stem and secretory cells to illustrate that CHEs likely arise from the secretory lineage. Furthermore, through pharmacological inhibition experiments, we demonstrated that Notch signaling is necessary for CHE cell fate specification both *in vivo* and *in vitro*.

These findings collectively suggest that Notch must be reactivated along the secretory lineage to drive CHE fate, likely via the induction of alternative receptors such as Notch2, and may have broader implications for the behavior and diversification of specialized secretory cells. In particular, this notion of Notch reactivation in CHEs challenges the prevailing model of the secretory lineage as Notch-off. Notably, this model of a wholly Notch-off secretory lineage has been informed by lineage tracing using mouse models (Yang et al., 2001), which do not generate a comparable CHE/BCHE population under homeostatic conditions. As rats and human models lack similar lineage-tracing capabilities, we cannot definitively rule out the possibility that CHEs could arise from an absorptive, Notch-on progenitor. Indeed, the loss of CHEs upon Notch inhibition we observed would also be consistent with this alternative hypothesis. Even when conventional genetic lineage-tracing techniques are available, however, it is challenging to appropriately evaluate the secretory lineage considering the comparative rarity and complexity of secretory cell types. Our evidence of Notch reactivation during CHE differentiation cautions against inferring the final downstream Notch signaling status in differentiated cell types from the initial Notch signaling status of their less determined progenitors. Alternative methods for transducing these signals can be temporally restricted to ensure an orderly transition in developmental potential. Ultimately, our results highlight the surprising modularity of the secretory lineage despite its restricted origin within the intestinal crypt.

We propose a working model of CHE cell differentiation in which: (1) CHEs arise from the secretory lineage, which is initially Notch-off, (2) Notch is reactivated along the secretory lineage as a potentially multipotent Notch2^+^/Prox1^+^ progenitor and (3) Meis1 is upregulated to promote the expression of differentiated CHE markers, including CFTR and Best4. While we and others (Burclaff et al., 2022) have computationally described CHEs and BCHEs respectively as arising from the secretory lineage, our work further identifies a resident Notch2^+^/Prox1^+^ progenitor in crypts that can divide symmetrically, suggesting that Notch reactivation may precede terminal CHE differentiation.

Beyond their localization to the crypt, undifferentiated Notch2^+^/Prox1^+^ cells generally lack Meis1, the only CHE-specific transcription factor identified in our scRNA-seq, but can occasionally be found with low levels of nuclear Meis1, which suggests the onset of CHE differentiation. Further investigation is needed to determine the effect of Meis1 on CHE fate. Additionally, the full lineage-generating capability of this Notch2^+^/Prox1^+^ progenitor *in vivo* remains to be tested and is currently limited by the lack of genetic tools available in rat and human models. Nevertheless, a previous mouse study characterized Prox1^+^ progenitors as having both reserve stem cell activity and rare clonogenic potential in homeostasis (Yan et al., 2017), features that our putative Notch2^+^/Prox1^+^ progenitor may share in the rat.

Collectively, these results highlight the value of exploring the secretory lineage beyond the characterization that has been done in mice. Furthermore, the increasing diversity of cell types that arise from the same secretory progenitor warrants detailed characterization of their gene regulatory networks, including the specific transcription factor-binding profiles and stepwise order of molecular events required to appropriately partition and define their unique functions. Intriguingly, our data demonstrate a high nuclear Hes1 intensity level in differentiated CHEs and considerably lower nuclear Hes1 levels in the stem/progenitor zone. Hes1 is notable for its oscillatory activity (Hirata et al., 2002), which regulates differentiation timing in a variety of systems (Seymour et al., 2020; Maeda et al., 2023). Since Hes1 oscillations have been described in intestinal stem cells (Weterings et al., 2024 preprint), it is possible that CHE specification may involve a transition from oscillatory Hes1 in stem cells, through a Notch-off secretory progenitor, and finally towards more sustained Hes1 expression in differentiated CHEs, although this remains to be explored.

The fact that CHEs are so highly enriched for Hes1 and require active Notch signaling for their fate specification is perhaps less surprising when considered alongside pulmonary ionocytes, a rare CFTR-enriched cell type within the lung that also requires Notch signaling for proper specification (Plasschaert et al., 2018). Although these cell types appear to be analogous, there is no evidence of Foxi1 (forkhead box I1) expression within any intestinal cell type (data not shown), even though this transcription factor is necessary and sufficient for pulmonary ionocyte fate (Plasschaert et al., 2018; Montoro et al., 2018) and is expressed within the ionocytes of many tissues (Pou Casellas et al., 2023). Notably, Foxi1 has also been described as promoting ionocyte, tuft cell and neuroendocrine cell specification through a shared rare Foxi1^+^ progenitor in developing airway epithelium (Yuan et al., 2023). Although these airway cell types exhibit highly similar transcriptional relationships to those we have found for CHEs, tuft cells and EECs within the intestinal secretory lineage, the total absence of Foxi1 within our small intestinal transcriptional atlas indicates that similar developmental relationships may hold despite an alternative transcriptional program. As a putative alternative, our results and the work of others (Yan et al., 2017) suggest that this unique secretory sub-branch may be driven more directly via Prox1, a possibility that warrants further characterization.

While ionocytes were initially described as a major source of CFTR activity and secretion in the lung (Plasschaert et al., 2018), subsequent work has clarified that the majority of CFTR expression and secretion is performed by other secretory cells, which vastly outnumber rare ionocytes (Okuda et al., 2021). We observed that DAPT-treated organoids, which exhibit decreased CHE specification, displayed a strong reduction of forskolin-induced swelling. However, DAPT is also known to reduce enterocytes, which also express CFTR, and therefore our studies cannot confirm a direct causation between the absence of CHEs and the abrogation of swelling. Supporting the notion that comparatively rare CHEs may be capable of major effects on organoid swelling, however, Wang et al. (2025), demonstrated in human intestinal organoids that swelling induced by the STa analog linaclotide is absent upon complete loss of CHEs induced by genetic ablation of the transcription factor SPIB. This suggests that, at least *in vitro*, CHEs may be a major source of secretion, although the secretory function for CHEs *in vivo* has yet to be confirmed. Our transcriptomic analysis suggests that *Spib* is highly enriched in both the CHE and tuft cell populations (Fig. S3M), supporting the common CHE/tuft fate trajectory we observed.

Ionocytes have also been hypothesized to be involved in the regional coordination of pH sensing and acute responses to hyperosmotic stress across a range of systems (Okuda et al., 2021; Yuan et al., 2023). It is possible that CHEs could similarly coordinate regionalized pH regulation in response to lumen acidification. *In vivo* intestinal-looping studies recently demonstrated that CHEs are capable of sensing and responding to a low pH environment by rapidly trafficking CFTR, guanylyl cyclase C and OTOP2 to their apical domains (dos Reis et al., 2025 preprint). CHEs are highly enriched for apical CFTR, and therefore capable of bicarbonate efflux out into the luminal environment (Garcia et al., 2009). Furthermore, CHEs are regionalized to the distal duodenum and proximal jejunum, regions of the intestine where residual stomach acid must be effectively neutralized to promote optimal nutrient absorption (Jakab et al., 2013). This putative function would be particularly relevant for cystic fibrosis, in which lumen acidification driven by the loss of CFTR-mediated bicarbonate secretion drives gastrointestinal pathophysiology and results in significant co-morbidities (Garcia et al., 2009; de Lisle and Borowitz, 2013). Further studies will be needed to determine the specific functional contribution of CHEs to the overall physiology of the small intestine in the context of homeostasis and disease.

## MATERIALS AND METHODS

### Model system and permissions

All studies were conducted with Institutional Animal Care and Use Committee (IACUC) approval from Yale University's Standing Committee on animals, and in accordance with institutional guidelines for the humane treatment of animals.

### Experimental animals

Male adult Sprague-Dawley rats (*Rattus norvegicus*) with an average weight of 200 g were purchased from Charles River. Rats were housed in the animal care facility of the Yale Animal Resources Center according to institutional guidelines for the care and use of laboratory animals.

### Cell dissociation and droplet-based sequencing

Epithelial cell suspensions were generated as described previously with modifications (Sumigray et al., 2018). Briefly, the proximal jejunum of adult male Sprague-Dawley rats was isolated and rinsed in ice-cold PBS. The tissue was segmented into ∼2 cm fragments and opened longitudinally to expose the epithelium. The tissue was incubated in 30 mM EDTA in HBSS (Gibco, 14170112) at 37°C for 20 min. The tissue was vigorously shaken to release epithelium. The epithelium was collected into a 15 ml conical tube, incubated on ice, and allowed to settle via gravity. The epithelium was washed twice in HBSS, then incubated in 1 mg/ml dispase II (Roche, GE-0619) in HBSS for 10-12 min at 37°C with frequent shaking. A total of 1 ml fetal bovine serum (FBS) and 2 µl DNase I was added to the cell suspension, and the cells were passed through 70 µm strainers. Cells were collected by centrifugation at 300 ***g*** for 5 min at 4°C. Cell pellets were washed with HBSS+10% FBS and centrifuged again at 300 ***g*** for 5 min at 4°C. Cells were resuspended in HBSS+10% FBS at a 1200 cells/µl. Prior to droplet-based scRNA-seq, cells were run through a 40 µm filter. Single-cell suspensions were processed through several additional rounds of purification, centrifugation and resuspension before the generation of an emulsion using the NextGem v.3.1 3′UTR capture kit (10x Genomics) with a target recovery of 10,000 cells. The resulting reverse transcription and generation of the sequencing library were performed according to the manufacturer's protocols. The final library was sequenced to a target recovery of 300 million read pairs and conducted at the Yale Center for Genomic Analysis.

### Analysis of rat scRNA-seq data

#### Pre-processing

Sequencing data were demultiplexed and processed using Cell Ranger v.5.0 with alignment to Rnor6, followed by clustering and expression analysis using Seurat v.4.0.1 package in R (v.4.0.3). Prior to downstream processing, 20,285 cells were recovered with a median transcript count of 32,331 unique molecular identifiers per cell. Cells with >200 and <5800 genes and >5000 transcripts were retained. Cells with >40% mitochondrial transcripts were excluded. Libraries from two separate rats were integrated. Data were normalized and log-transformed using the ‘LogNormalize’ function in Seurat. All clusters expressing *Cd45* (*Ptprc*) or hemoglobin (*Hba-a1*) were removed. This left a total of 7362 cells, confirmed to be epithelial by *Epcam* expression. Clusters were assigned to known cell types by signature gene markers, as outlined in Table S1.

#### Dimensionality reduction and clustering

The top 2000 variable genes were used for principal component analysis, and the top 20 principal components were selected and visualized by uniform manifold approximation and projection (UMAP). We identified clusters using the ‘FindClusters’ function in Seurat, using a parameter resolution of 0.3. As mentioned above, clusters enriched for *Ptprc* or *Hba-a1* were removed, and data were rescaled. The ‘FindNeighbors’ and ‘FindClusters’ functions were rerun, with 20 principal components and a resolution of 0.5. The ‘AddModule Score’ function in Seurat was used to generate scores for cell-type markers and for Notch-on and Notch-off’ scores. The genes that contributed to each score are listed in Table S1.

### Rat intestinal organoid derivation and cell culture

Rat intestinal organoids were established from the crypts of rat proximal jejunum as described previously (Zagoren et al., 2023). Briefly, following euthanasia, rat proximal jejunum was isolated and opened longitudinally to expose villi, which were scraped off with a glass microscope slide. Intestinal fragments were incubated for 30 min in 3 mM EDTA at 4°C, after which the intestinal segment was shaken vigorously in PBS with fine tweezers under a dissecting microscope until the PBS primarily contained crypts. Crypts were pelleted at 250 ***g*** and the crypt pellet was added to growth factor-reduced Matrigel. Rat intestinal organoids were then cultured in rat intestinal organoid media (Zagoren et al., 2023) at 37°C, 5% CO_2_, and passaged by mechanical disruption every 3-5 days as needed.

### Immunofluorescence staining

For cryosections, 10-µm-thick sections of rat proximal jejunum or distal duodenum were fixed in 4% paraformaldehyde (PFA) in PBS with 0.2% Triton X-100 (PBST-0.2%) (American Bio, AB02025) for 8 min. Tissues were placed into block [3% bovine serum albumin (Sigma-Aldrich, A9647); 5% normal donkey serum (Sigma-Aldrich, D9663) in PBST-0.2%] for 1 h. Primary antibody was then added for 15 min to overnight, depending on the antibody. After washing three times with PBST-0.2%, sections were incubated in secondary antibody for 10 min at room temperature. Sections were then washed three additional times in PBST-0.2%, after which they were mounted in Antifade and imaged on an upright Zeiss AxioImager with Apotome 2 attachment with a Zeiss AxioCam 506 mono camera. Objectives used were Plan Apochromat 10×/0.45 air, 20×/0.8 air, 40×/1.3 oil and 63×/1.4 oil.

Whole-mount staining of rat jejunum was performed as described previously (Hu et al., 2022). Briefly, following euthanasia, rat proximal jejunum was isolated, opened longitudinally to expose villi, and fixed in 100% ice-cold methanol (Fisher Scientific, 02-003-351). Tissues were subsequently permeabilized for 6 h in PBS with 0.3% Triton X-100 (PBST-0.3%), incubated with primary antibody for 16 h, washed five times with PBST-0.3% for 1 h each wash, incubated with secondary antibody for 16 h, washed ten times with PBST-0.3% for 30 min each wash, and then post-fixed overnight in 4% PFA/PBS. Samples were then mounted in Ce3D media and imaged on a Leica Stellaris 5 confocal laser scanning microscope with a white light laser using a HC FLUOTAR 25×/0.95 W VISIR objective or HC PL APO 40×/1.10 W CORR CS2 objective (Leica, Germany).

For whole-mount staining of organoids, organoids were fixed in 4% PFA in PBST-0.2% in a cell culture dish for 10 min. Organoids were released from the Matrigel by pipetting up and down, collected in a 1.5 ml tube, and PFA was removed and replaced with PBST-0.2%. After allowing organoids to settle, the pellet was resuspended in block and incubated at room temperature for 45 min. Primary antibody incubation was carried out at room temperature for 45 min to overnight, depending on the antibody. Organoids were then washed quickly five times with a transfer pipette, allowing the organoids to settle to the bottom of the tube prior to each wash, followed by a 5 min incubation in PBST-0.2% at room temperature, nutating. This process was repeated three times. Secondary antibody was added for 30 min at room temperature. Organoids were again washed as above in PBST-0.2%. Lastly, organoids were mounted in Antifade and imaged on a Leica Stellaris 5 confocal using the settings described above.

The primary antibodies used were as follows. Rabbit anti-CFTR antibody, used 1:500 overnight, was described previously (Golin-Bisello et al., 2005). Rabbit anti-Best4 antibody, used 1:250 overnight, is a custom rabbit polyclonal antibody (described below). The remaining primary antibodies are commercially available: mouse anti-Meis1 (1:100 overnight; Invitrogen, MA5-27191), mouse anti-Prox1 (1:100 overnight; Novus Biologicals, NBP1-30045), goat anti-Notch2 (1:100 15 min; R&D Systems, AF1190-SP), mouse anti-Atoh1 (1:40 overnight; Developmental Studies Hybridoma Bank), rabbit anti-Muc2 (1:1000 overnight; Abcam, ab272692), rabbit anti-Dclk1 (1:1000 overnight; Abcam, ab31704), rabbit anti-ChgA (requires prefixation, 1:500 overnight; Abcam, ab254557), sheep anti-Dll1 (1:100 overnight; R&D Systems, AF5026-SP), rabbit anti-Ki67 (1:200, 1 h; Abcam, ab15580), rabbit anti-phospho-histone3 (1:500, 1 h; Cell Signaling Technology, 3377), rabbit anti-Notch1 (1:100 15 min; Cell Signaling Technology, 3808S), rabbit anti-Hes1 (1:2500; Cell Signaling Technology, 11988S). Note that the Hes1 antibody requires a 7.5 min incubation with Tyramide Signal Amplification Kit (Thermo Fisher Scientific, B40922).

Secondary antibodies were used at 1:200 as follows: donkey anti-goat Alexa Fluor 488 (Jackson ImmunoResearch, 705-545-147), donkey anti-mouse Alexa Fluor 488 (Jackson ImmunoResearch, 715-545-151), donkey anti-rabbit Alexa Fluor 488 (Jackson ImmunoResearch; 711-545-152), donkey anti-goat Alexa Fluor 647 (Jackson ImmunoResearch, 705-605-147), donkey anti-mouse Alexa Fluor 647 (Jackson ImmunoResearch, 715-605-151), donkey anti-rabbit Alexa Fluor 647 (Jackson ImmunoResearch, 711-605-152), phalloidin Alexa Fluor 647 (Invitrogen, A22287), donkey anti-goat Rhodamine Red (RRX) (Jackson ImmunoResearch, 705-295-147), donkey anti-mouse RRX (Jackson ImmunoResearch, 715-295-151), donkey anti-rabbit RRX (Jackson ImmunoResearch, 711-295-152), donkey anti-sheep RRX (Jackson ImmunoResearch, 713-295-147). DAPI (5 µg/ml; Invitrogen, D1306) was used as nuclear stain.

For co-labeling of Best4 and CFTR, FlexAble Antibody labeling kits CoraLite Plus 488 and CoraLite Plus 555 (Proteintech) were used according to the manufacturer's protocols.

### Best4 antibody generation

The custom Best4 polyclonal antibody was generated by GenScript against rat Best4 290-454 aa. The above protein fragment was tagged with 6X His as antigen and used to immunize two rabbits, followed by affinity purification from each rabbit. The antigen sequence was as follows: M**HHHHHH**LKVAEQLINPFGEDDDDFETNQLIDRNFQVSLLSVDDMYQNLPPTEQDLYWDEARPQPPYTVATAAESQRPSFMGSTFNLRMSDDPEQCLQVEASRRFDLPAPVTQTSQTPLLGRFLAARAPSPAVSLQNLRTSHLLHLRPGGEEAEGRIEEAVDEGSENEAMEP. Each antibody was confirmed by GenScript to bind to the target protein immunogen by western blotting, to have an ELISA titer of >1:128,000 and to not react with total IgG negative control. Staining patterns of each custom Best4 antibody in the intestine were compared against commercially available Best4 antibody staining (Novus Biologicals, NB110-55612) in combination with the CHE markers used in this study.

### Treatment with DAPT

Adult male Sprague-Dawley rats (*n*=3 for DAPT treatment, *n*=3 for DMSO control) were injected with 20 mg/kg DAPT or an equivalent volume of DMSO once daily for five consecutive days. At day 6, rats were euthanized in accordance with IACUC-approved institutional guidelines. The intestine was dissected following a previously described protocol (Zagoren et al., 2023) and distal duodenum and proximal jejunum were frozen in TissueTek OCT Compound (Sakura, 4583) for cryosectioning.

For organoids, DAPT powder (Millipore, 565770) was dissolved in DMSO to a stock concentration of 10 mM. Rat jejunum organoids were incubated in 5 µM DAPT or an equivalent volume of DMSO control for 3 days at 37°C, 5% CO_2_.

### Forskolin swelling of organoids

Differentiated rat intestinal organoids (∼2-3 days post-passage) were cultured in rat intestinal organoid media in 35 mm glass-bottom dishes. For treated organoids, forskolin (Sigma-Aldrich, F3917), was diluted to a 10 µM working concentration, whereas for control organoids an equivalent volume of DMSO (vehicle) was added. Swelling was imaged every 10 min for 120 min total using the brightfield setting of a Leica Stellaris 5 confocal microscope.

### Use of artificial intelligence tools

To count large numbers of epithelial cell nuclei, mesenchymal cells were manually removed in Fiji, and epithelial-only images were imported into AIVIA software (Leica). The ‘Cell Count/ CellPose’ feature was then modified to precisely detect and quantify epithelial nuclei. All artificial intelligence counting was manually confirmed by eye to ensure cells were adequately captured and represented.

### Statistical analysis

All results are expressed as mean±s.d. The significance of the differences between different treatments and time points was compared using a two-tailed unpaired *t*-test in Prism 9 (GraphPad) unless noted otherwise in the figure legend. *P*-values less than 0.05 (*P*<0.05) were considered statistically significant. For all plots, error bars indicate s.d.

## Supplementary Material



10.1242/develop.204277_sup1 Supplementary information
