# Supplementary material for: Evidence of secondary Notch signaling within the rat small intestine
Source: Development. 2025 Jun 3;152(11):dev204277. doi: 10.1242/dev.204277 (PMC12188240; doi:10.1242/dev.204277)
Supplement: Supplementary information [file develop-152-204277-s1.pdf]

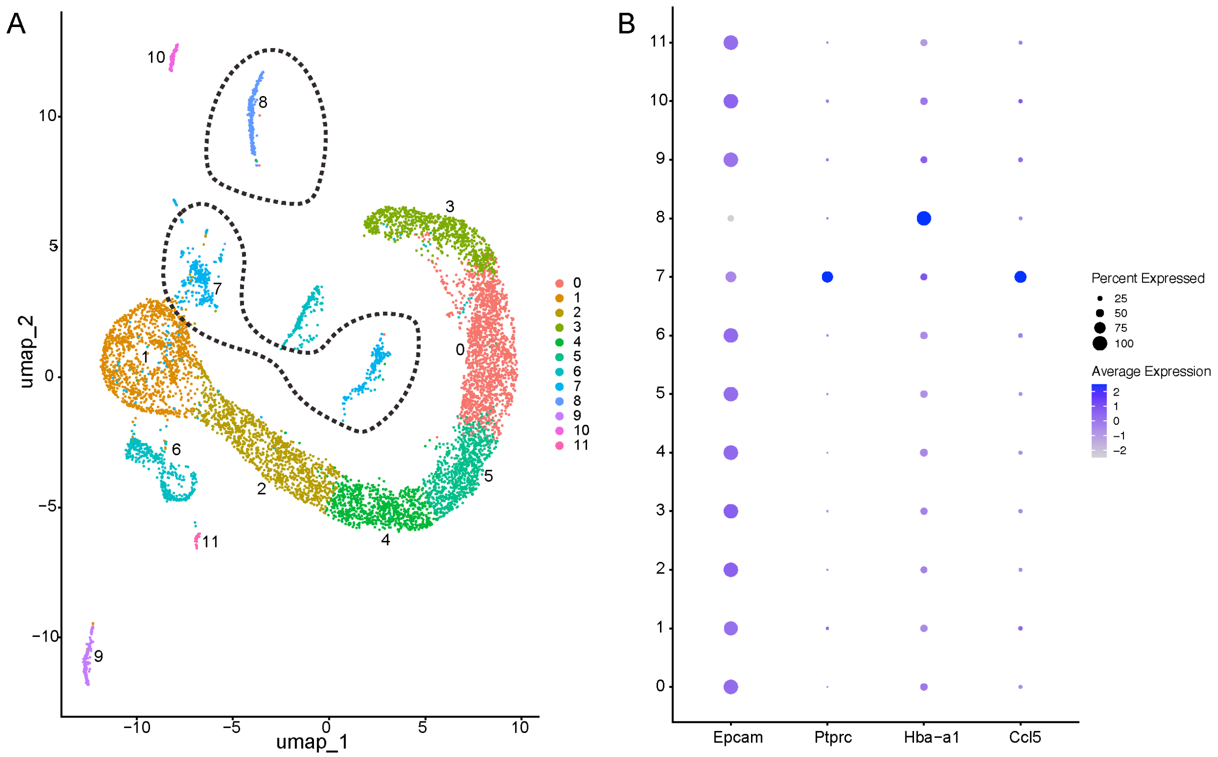

Fig. S1. Removal of Non-Epithelial Clusters

A) UMAP of single-cell RNAseq data from rat intestinal jejunum showing initial unannotated clustering prior to filtering out non-epithelial cells. Clusters 7&8 (non-epithelial) are highlighted with dashed lines. B) Clusters were analyzed for expression of epithelial (Epcam), immune (Ptprc, Ccl5), and blood markers (Hb1a1). Clusters expressing immune and blood markers that were negative for Epcam were removed (i.e. clusters 7&8).

Development • Supplementary information

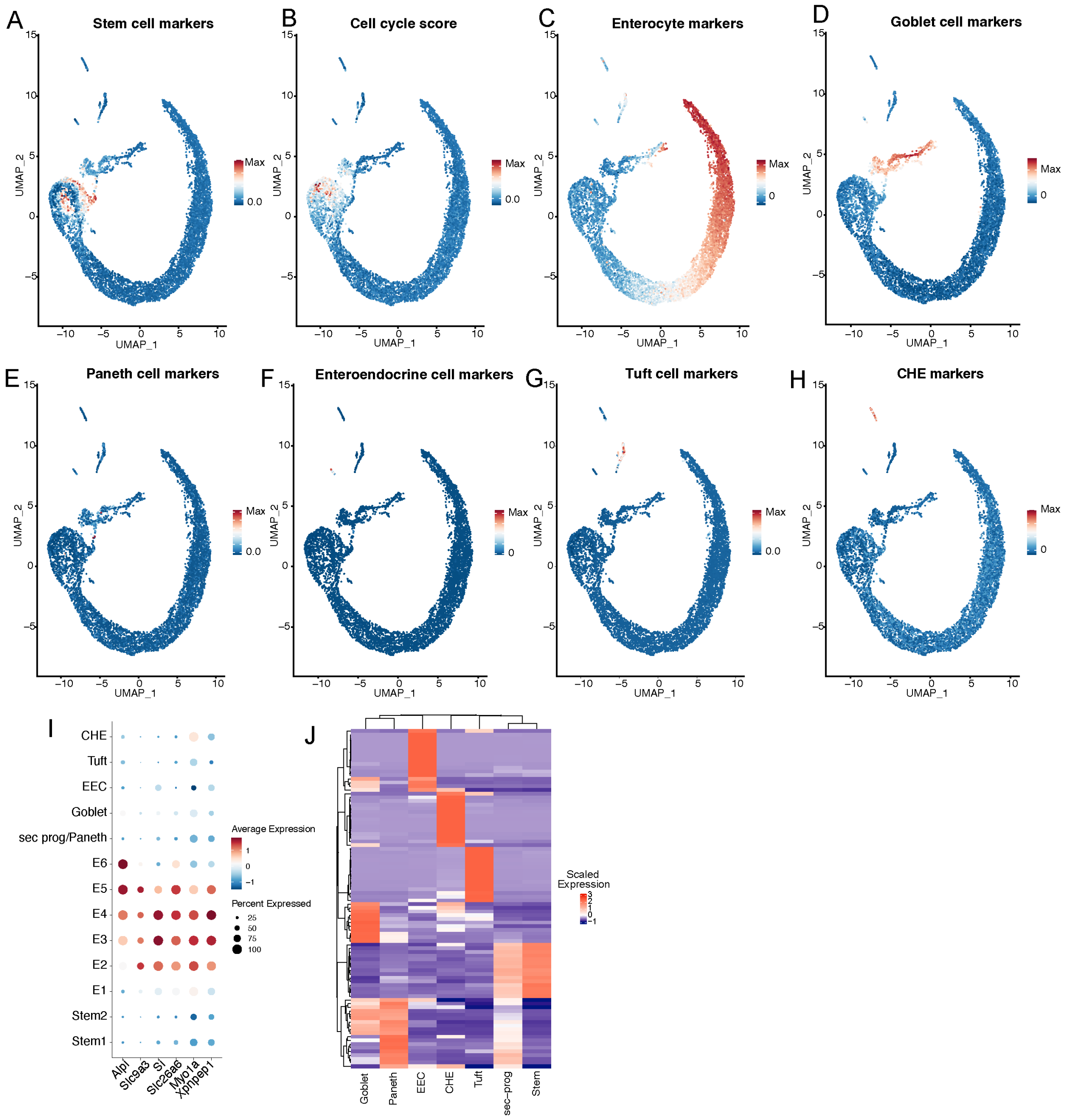

Fig. S2. Cluster identification by marker gene expression

A-H) UMAP of single cell RNA-seq data from rat intestinal jejunum color-coded by expression of A) stem cell markers, B) cell cycle score, C) enterocyte markers, D) Goblet cell markers, E) Paneth cell markers, F) EEC markers, G) Tuft cell markers, H) CHE markers. Marker genes are listed in Supp. Table 1. I) Dotplot for expression of differentiated cell type markers and enterocyte-associated genes across intestinal epithelial cell types. CHEs did not express markers of any other differentiated cell types or enterocyte-associated genes. J) Heatmap illustrating cell-type specific characteristic gene expression patterns.

Development • Supplementary information

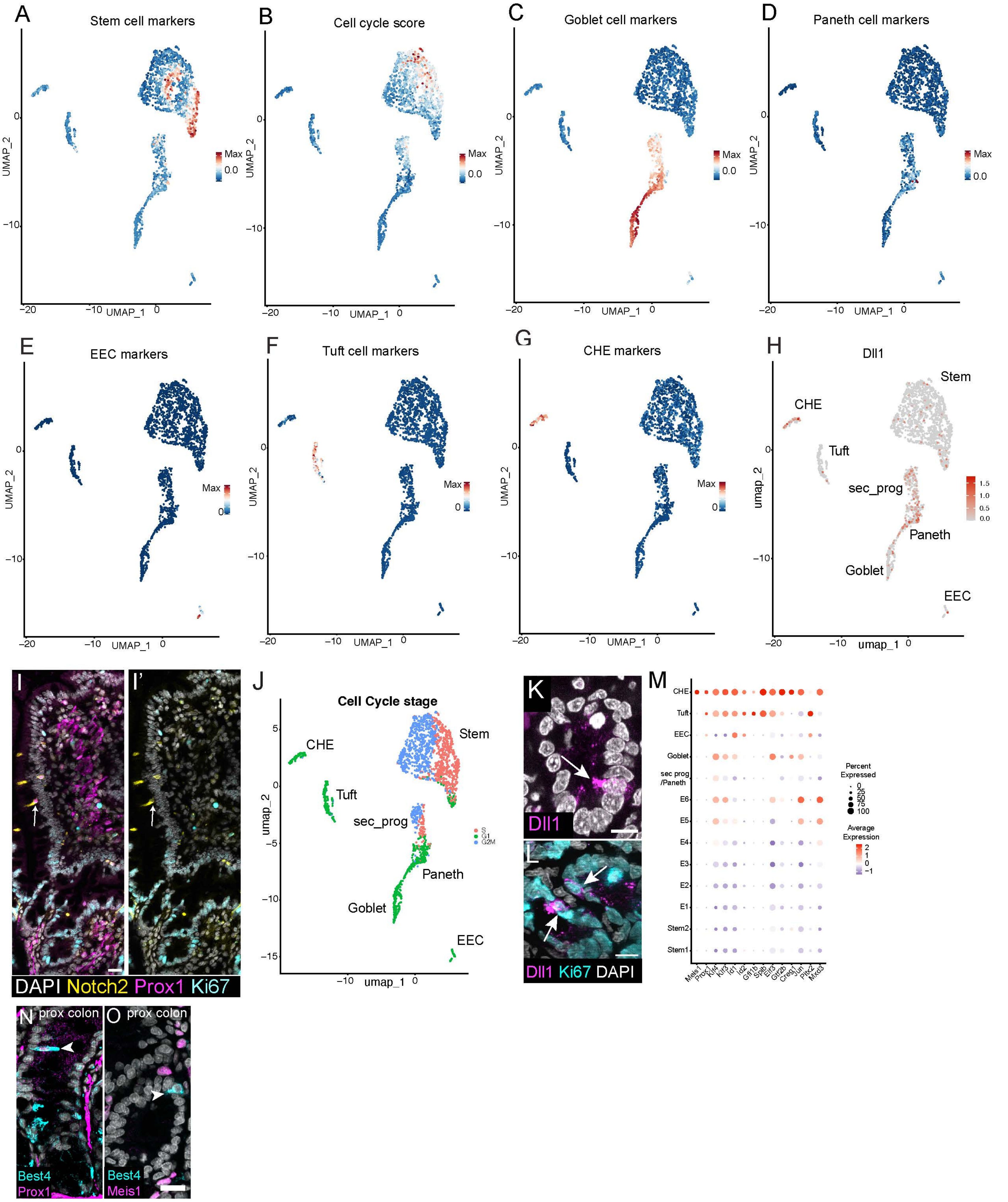
A
Stem cell markers
B
Cell cycle score
C
Goblet cell markers
D
Paneth cell markers
E
EEC markers
F
Tuft cell markers
G
CHE markers
H
Dll1
Stem
CHE
Tuft
sec_prog
Paneth
Goblet
EEC
UMAP_1
UMAP_2
umap_1
umap_2
Max
0.0
I
I'
DAPI Notch2 Prox1 Ki67
J
Cell Cycle stage
S
G1
G2M
K
Dll1
L
Dll1 Ki67 DAPI
M
CHE
Tuft
EEC
Goblet
sec prog /Paneth
E6
E5
E4
E3
E2
E1
Stem2
Stem1
Percent Expressed
Average Expression
Meis1
Prox1
Klf4
Klf3
Id1
Id2
Gfi1b
Spib
Elf3
Gfi2b
Creg1
Jun
Pitx2
Mxd3
N prox colon
Best4
Prox1
O prox colon
Best4
Meis1

Fig. S3. Secretory cell cluster characterization

A-G) UMAP of reclustered stem and secretory progenitors color-coded by expression of A) stem cell markers, B) cell-cycle score, C) Goblet cell markers, D) Paneth cell markers, E) EEC markers, F) Tuft cell markers, G) CHE markers. Marker genes are listed in Supp. Table 1. H) UMAP of stem and secretory cell clusters color-coded by Dll1 expression. I,I') Differentiated CHEs in villi (arrow) marked by Notch2 (yellow) and Prox1(magenta) are not positive for Ki67 (cyan). J) Stem and secretory cell UMAP annotated by cell cycle stage: S-phase (red), G1 (green), G2/M (blue). K) Secretory progenitor in rat proximal jejunum, occupying the +4 position and expressing high levels of membrane-localized Dll1 (magenta). DAPI in white. Scale bar, 10 μm. L) Dll1 (magenta) and Ki67 (cyan) costaining of +4 cells. Scale bar, 10 μm. M) Dotplot from scRNA-seq data of expression of transcription factors enriched in CHEs. N) Best4+ colon cells (Best4, cyan) were Prox1+ (magenta) in rat proximal colon. DAPI in white. O) Meis1 (magenta) was undetectable in Best4+ colon cells (Best4, cyan). DAPI in white. Scale bar for M-N, 20 μm.

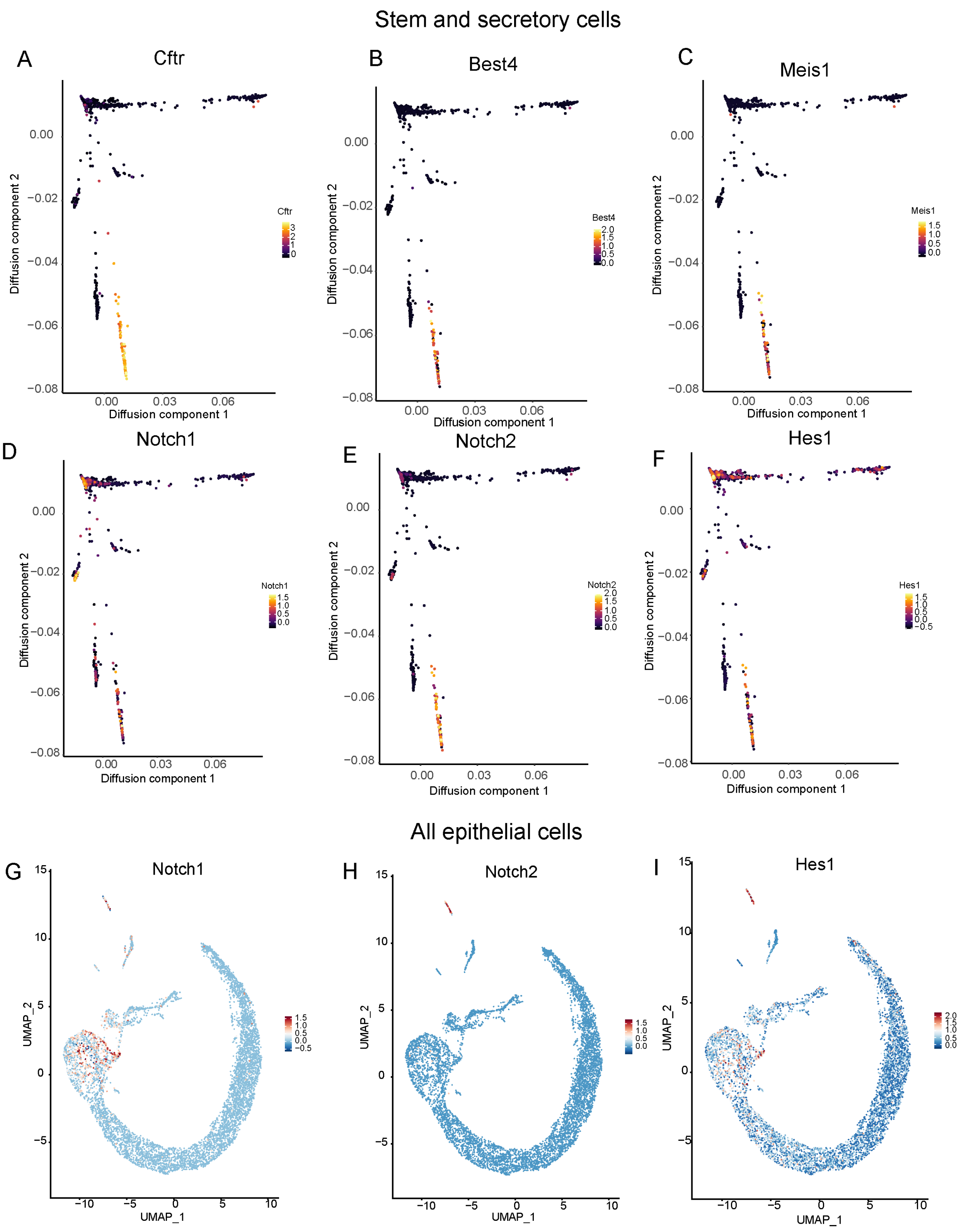

Fig. S4. Secretory lineage characterization across pseudotime

A-F) Diffusion maps of stem cells and secretory progenitors as a readout of pseudotime. Note the clear bifurcation between Goblet/Paneth and the EEC/Tuft/CHE lineages. Diffusion map color coded for A) Cftr expression, B) Best4 expression, C) Meis1 expression, D) Notch1 expression, E) Notch2 expression, and F) Hes1 expression.

Development • Supplementary information

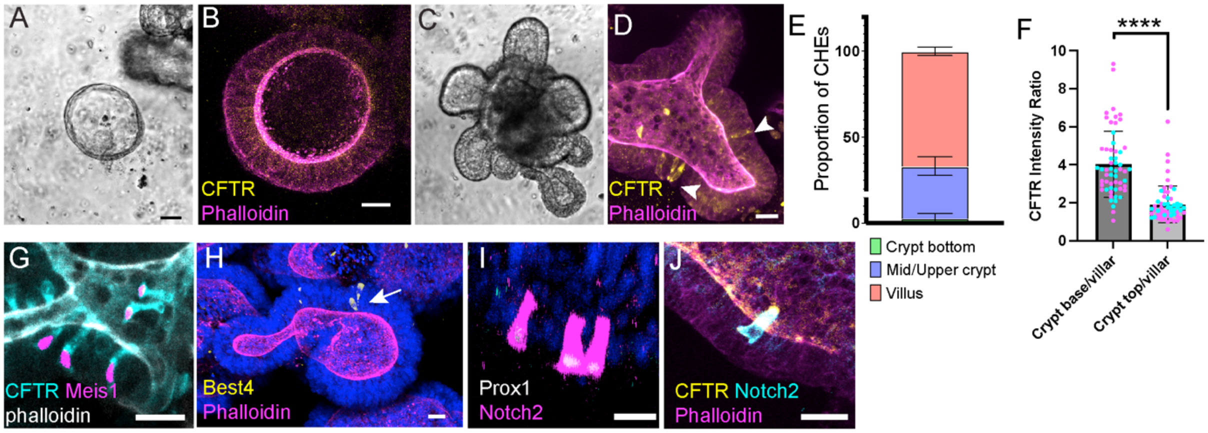

Fig. S5. Rat intestinal organoids generate CHEs

A) Undifferentiated rat jejunum organoid form spheroids. Scale bar, 50 μm. B) CFTR (yellow) in undifferentiated rat intestinal organoids. Phalloidin in magenta. Scale bar, 20 μm. C) Differentiated rat intestinal organoid ~3 days post-passaging. Scale bar in panel A. D) Rat organoids differentiate to form CHE cells. CFTR (yellow), phalloidin (magenta). Scale bar, 20 μm. E) Distribution of CHEs in organoids recapitulates *in vivo* distribution. N = 3 technical replicates from two independently derived organoid lines. F) CFTR intensity in organoids recapitulates *in vivo* distribution. N = 3 technical replicates from two independently derived organoid lines. Two-tailed paired t test. $p<0.0001$. G-J) CHE in organoids also expressed Meis1, Prox1, Best4, and Notch2 as seen *in vivo*. G) CHEs with CFTR (cyan) and Meis1 (magenta). H) Best4+ cells (yellow) in organoids. Phalloidin in magenta. I) CHEs that are Prox1+ (white) and Notch2+ (magenta). J) CHEs that are CFTR+ (yellow) and Notch2+ (cyan). Scale bars, 20 μm.

Development • Supplementary information

Table S1. Genes used to generate module scores for cell cluster identification and Notch signaling scores.

**Module Score**	**Gene**
Stem cell	Lgr5, Ascl2, Slc12a2, Axin2, Olfm4, Gkn3
Cell cycle	Mki67, Cdk4, Mcm5, Mcm6, Pcna
Enterocyte	Alpi, Apoa1, Apoa4, Fabp1
Goblet	AABR07006030.1, Tff3, Agr2
Paneth	Lyz2, Wnt3, Dll4, Defa24, Wnt11
Enteroendocrine	Chga, Chgb, Tac1, Tph1, Neurog3
Tuft	Dclk1, Trmp5, Gfi1b, Il25
CHE	Cftr, Best4
Notch on	Notch1, Notch2, Notch3, Notch4, Hes1, Nrarp
Notch off	Dll1, Dll4, Jag1, Jag2, Atoh1
