# Supplementary material for: Evidence of secondary Notch signaling within the rat small intestine
Source: Development. 2025 Jun 3;152(11):dev204277. doi: 10.1242/dev.204277 (PMC12188240; doi:10.1242/dev.204277)
Supplement: Supplementary file 1 [file develop-152-204277_review_history.pdf]

Evidence of secondary Notch signaling within the rat small intestine

Eleanor Zagoren, Nicolas Dias, Anderson K. Santos, Zachary D. Smith, Nadia A. Ameen and Kaelyn Sumigray
DOI: 10.1242/dev.204277

Editor: James M. Wells

Review timeline

Original submission:	24 July 2024
Editorial decision:	10 September 2024
First revision received:	17 March 2025
Editorial decision:	21 April 2025
Second revision received:	25 April 2025
Accepted:	28 April 2025

Original submission

First decision letter

MS ID#: dev.204277

MS TITLE: A second wave of Notch signaling diversifies the intestinal secretory lineage

AUTHORS: Eleanor Zagoren, Nicolas Dias, Zachary D. Smith, Nadia A. Ameen and Kaelyn Sumigray

Dear Dr Sumigray,

I have now received all the referees' reports on the above manuscript, and have reached a decision. The referees' comments are appended below, or you can access them online: please go to:

As you will see, the referees express considerable interest in your work, but have some significant criticisms and recommend a substantial revision of your manuscript before we can consider publication. If you are able to revise the manuscript along the lines suggested, which may involve further experiments, I will be happy receive a revised version of the manuscript. Your revised paper will be re-reviewed by one or more of the original referees, and acceptance of your manuscript will depend on your addressing satisfactorily the reviewers' major concerns. Please also note that Development will normally permit only one round of major revision. If it would be helpful, you are welcome to contact us to discuss your revision in greater detail. Please send us a point-by-point response indicating your plans for addressing the referees’ comments, and we will look over this and provide further guidance.

Please attend to all of the reviewers' comments and ensure that you clearly highlight all changes made in the revised manuscript. Please avoid using 'Tracked changes' in Word files as these are lost in PDF conversion. I should be grateful if you would also provide a point-by-point response detailing how you have dealt with the points raised by the reviewers in the 'Response to Reviewers' box. If you do not agree with any of their criticisms or suggestions please explain clearly why this is so.

Reviewer 1

Advance summary and potential significance to field

© 2025. Published by The Company of Biologists under the terms of the Creative Commons Attribution License (https://creativecommons.org/licenses/by/4.0/).

This is a strong paper that utilizes sophisticated computational tools to identify the species-specific identity of CHE cells. The single cell data are very nicely supported by immunofluorescence data. Overall, it is a well-done study. A few suggestions below:
1) It would be helpful if possible to show mouse and rat in the same crypt to villus axis; much of the villus portion in the mouse is missing. It might be difficult to get more rat tissue but for mouse tissue this should be doable. Also, it would be clearer if DAPI were the same color in both panels and labelled in the figure.
2) Was CFTR expression in mesenchyme region expected? The authors might want to comment on this.
3) The number of CHE positive cells seems a bit small - it would be worth mentioning in the text how many cells you were able to isolate. Given the possible expression in the mesenchyme, it might be worth showing that these cells are EPCAM positive somewhere in the scRNA seq data [unless it was filtered for EPCAM].
4) Can the authors quantify the number of BEST+ cells from proximal jejunum to ileum/colon?
5) Figure 2 results are very nice, and the only minor suggestion is in Figure 2B to move the letters from on top the clusters like in other figures. Also to capitalize the word sec_prog so it matches the rest. It might also be worth on the top of Figure 2B to label this "reclustered cells" or circle in 2A which cell types were reclustered for 2B.
6) In Figure 3, the expression of Notch 2 in the single cell data is confusing. The immunofluorescence very nicely supports the presence of various Notch2+ Best4+/- and Notch2+ CFTR+/-, but it would be nice to see this in the single cell data. The Notch-on score is presented, but what about showing expression of individual genes in the pathway, overlayed with expression of Meis1, CFTR, and Best4?
7) Figure 4 has a lot of important data, especially the in vivo experiments and the organoid experiments. What is missing from this figure are descriptions of the experiment being done ie. DAPT injection in vivo, isolation of organoids and then adding DAPT in vitro. That would make this figure much easier to understand. Again DAPI is not labeled.
8) It could be helpful to explain a bit more to the reader about the purpose of the study and why rats were chosen as a model organism. This is currently scattered through the manuscript, and it could be consolidated.

Reviewer 2

Advance summary and potential significance to field

This manuscript focuses on uncovering the lineage development of Best4+ cells in the rat intestine. The authors identify these cells as CFTR High Expressors (CHEs), and use scRNAseq, immunohistochemistry, and organoid studies to argue that a "second wave of Notch signaling diversifies the intestinal secretory lineages" to form CHEs. As Best4+ cells are a relatively new intestinal cell type this study is of interest to the field as it describes these cells in detail. Because CHE cells are not found in the mouse, the rat is the most experimentally tractable in vivo model to understand the origin and function of these cells. The study is also of interest because the findings differ from the dominant view from human studies that Best4+ cells are absorptive lineage cells rather than secretory lineage cells. However, although interesting and provocative, the current study does not convincingly support the central theme of the manuscript concluding that CHE cells are secretory lineage cells requiring Notch signaling to be formed. Issues include over reliance on scRNAseq diffusion analysis and immunohistochemistry to suggest that CHE cells are part of the secretory lineage. Further, the results of the Notch pathway inhibition studies suggest that these cells might better fit with an absorptive cell lineage assignment. Thus, although an interesting topic with new information about marker expression in these cells, the studies do not definitively resolve developmental mechanisms for intestinal CHE cell differentiation.

Comments for the author

1. The assignment of CHE cells to the secretory lineage is largely based on scRNAseq studies. The diffusion analysis, which is a fundamental part of this study, does not convincingly suggest secretory lineage identify because the CHE cells are disconnected from secretory progenitors in the diffusion plots. Further, interpretations are based on very few cells CHE cells in the scRNAseq

© 2025. Published by The Company of Biologists under the terms of the Creative Commons Attribution License (https://creativecommons.org/licenses/by/4.0/).

dataset. Small cell numbers make predicted lineage connections less secure. Nevertheless, whatever the connection, bioinformatics analysis only promotes a hypothesis that would need to be supported by experimental data. The bioinformatics analysis is not in itself conclusive. And, because the findings contrast with current thinking in the field that these cells are in the absorptive lineage, the experimental data to support this claim need to be more definitive.

2. The Notch inhibition studies are quite interesting. Both in vivo rat studies and in vitro intestinal organoid studies suggested that CHE cell numbers are reduced when Notch is inhibited by treatment with a gamma secretase (GSI) inhibitor. Notch has been well described to regulate absorptive vs. secretory lineage differentiation in the intestine and GSI treatment promotes secretory cell differentiation and inhibits absorptive cell differentiation. Thus, the findings of this study better align with an absorptive lineage assignment, counter to the proposed secretory lineage assignment of the scRNAseq analysis and the overall conclusion of the study. A switch from Notch off to Notch on for a secretory cell would be of great interest. However, further studies are needed to support the hypothesis that there is a switch in Notch signaling after commitment to the secretory lineage to promote CHE cell differentiation via Notch signaling.

3. There are concerns about experimental rigor. Key studies are based on N=2. For example, the DAPT rat treatment studies do not have statistical power to make definitive conclusions about changes in cell numbers because only 2 rats were included in the analysis. Further, the data suggest incomplete Notch inhibition because the goblet cell hyperplasia was quite modest.

4. The histological analysis of marker expression used to map the expression pattern of proposed CHE lineage cells used sections that are poorly oriented. The sections should show complete crypt-villus cross sections to better support the proposed differentiation process. Further, generalized conclusions are made from high-powered images containing few cells. A more quantitative, morphometric approach is needed to solidify the conclusions of the immunostaining to quantify the various cell states along the crypt-villus axis.

5. The Notch 2 staining pattern is somewhat unexpected which questions the effectiveness of the antibody staining. Other studies would predict that Notch2 would be broadly expressed in absorptive lineage TA cells rather than restricted to a subset of secretory cells. Does the frequency and pattern of antibody stain match Notch2 mRNA expression in the RNAseq data set (note Fig3A does not include the absorptive lineage cells)? Moreover, Notch2 receptor expression does not necessarily indicate active Notch signaling. Is nuclear receptor (NICD) staining observed to support active Notch signaling in CHE cells or proposed CHE precursors?

6. Organoid studies could provide a system to probe the mechanism to support the proposed conclusions. Can the organoid studies be extended to support the proposed Notch off - Notch on mechanism for CHE differentiation?

7. The organoid forskolin secretion experiments are interesting, but do not directly link secretion to CHE cells. DAPT compromises organoid growth and changes differentiation of many different cell types, not just CHE cells.

8. The Discussion includes several speculative statements that are not supported by the study. Some examples are listed below.

Page 17 states that "Meis1 likely plays a key role in regulating small intestinal CHE fate, potentially as a terminal differentiation factor capable of upregulating the expression of ion channels required for luminal secretion such as CFTR and Best4."

Page 18 "The behavior of Hes1 between stem/progenitor cells and CHE may represent a transition from oscillatory to sustained Hes1 expression during CHE differentiation."

Page 19 "CHEs may similarly coordinate a regionalized pH regulation in response to lumen acidification."

Reviewer 3

Advance summary and potential significance to field

© 2025. Published by The Company of Biologists under the terms of the Creative Commons Attribution License (https://creativecommons.org/licenses/by/4.0/).

This study used single-cell RNA-seq data from rat intestine to investigate CFTR High Expresser cells (CHEs). These cells are also known in the human intestine as Best4+ cells. They are thought to be derived from the secretory lineage, and are of interest because their functions are still not well-characterized.
The authors investigated transcription factors enriched in this lineage and found that unlike the other secretory cell types like Goblet/Paneth cells, Atoh1 was not expressed in CHEs. Thus, the authors argued that Notch signaling is required in the differentiation of CHEs from the secretory progenitor.

1. The authors asserted that Notch2 is a candidate marker of CHE precursors (Page 10). CHEs are also the only cells expressing high Notch2 (also Page 10). Furthermore, the authors concluded that Notch must be reactivated to specify CHE cells (Page 15). This is an interesting idea that could be explored further by using Notch2 knock-out organoids to see whether they contain CHE cells. This would be a relatively straightforward experiment that can show whether Notch2 is necessary for CHE differentiation.

2. The authors performed Forskolin-mediated organoid swelling assay on DAPT-treated organoid, showing that organoids treated with DAPT underwent less swelling (Figure 4K-M). The authors then argued that the difference in swelling can be explained by the lack of CHE cells within organoids treated with DAPT (Page 14). However, considering that even in the authors' data CHE cells only comprise about 1-2% of the cells within the organoid (Figure 4F, J), it is difficult to believe that this change is seen due to the change in composition of the CHE cells.

Considering that enterocytes also express CFTR (as seen in public scRNA-seq data), and that enterocytes are the most abundant in the intestine, what is more likely is that the addition of DAPT reduced the number of enterocytes within the organoids, which in turn affect the swelling of the organoids. Ultimately, the authors did not show in a direct manner that CHE cells were the cause of swelling abrogation.

First revision

<u>Author response to reviewers' comments</u>

We thank the reviewers for their positive feedback and interest in this study. We performed additional experiments and clarified text as suggested by the reviewers, and point-by-point responses are listed below. There is discrepancy in the field of whether CHEs/Best4+ cells arise from the secretory or absorptive lineage. This is confounded by computational scRNA-seq data from human, where it has been suggested that the small intestinal Best4+ cells arise from the secretory lineage but the colonic Best4+ cells arise from the absorptive lineage (Burclaff et al, *CMGH*, 2022). Our data for the rat small intestine are consistent with this analysis and with that of zebrafish Best4+ cells that express a strong signature of secretory intestinal cells during their initial development (Sur et al, *Dev Cell*, 2023). While the absence of lineage tracing tools in rats precludes definitively identifying the origin of CHEs, the field is converging on CHEs falling within secretory cell specification pathways.

Reviewer 1: This is a strong paper that utilizes sophisticated computational tools to identify the species-specific identity of CHE cells. The single cell data are very nicely supported by immunofluorescence data. Overall, it is a well-done study. A few suggestions below:

We thank the reviewers for their strong support.

1) It would be helpful if possible to show mouse and rat in the same crypt to villus axis; much of the villus portion in the mouse is missing. It might be difficult to get more rat tissue but for mouse tissue this should be doable. Also, it would be clearer if DAPI were the same color in both panels and labelled in the figure.

© 2025. Published by The Company of Biologists under the terms of the Creative Commons Attribution License (https://creativecommons.org/licenses/by/4.0/).

Thank you for the suggestion. We have replaced the panels in Figure 1 that more clearly demonstrate the differences in CFTR expression and CHE localization along the entire crypt villus axis.

2) Was CFTR expression in mesenchyme region expected? The authors might want to comment on this.

In the images presented in Figure 1, the mesenchymal signal is autofluorescence from immune cells. We have now indicated this with asterisks and a description in the figure legend. We have also included here an image of CFTR WT vs. CFTR KO intestine (a kind gift from Susan Birket), and the stromal signal that the reviewer is referring to is preserved in the KO, while the epithelial signal is gone.
NOTE: We have removed unpublished data that had been provided for the referees in confidence.

3) The number of CHE positive cells seems a bit small - it would be worth mentioning in the text how many cells you were able to isolate. Given the possible expression in the mesenchyme, it might be worth showing that these cells are EPCAM positive somewhere in the scRNA seq data [unless it was filtered for EPCAM].

We have added the number of CHEs captured in the scRNA-seq experiment (88). We agree that it was indeed lower than expected. However, other studies have demonstrated that “fragile” cells like pulmonary ionocytes are less well represented in scRNA-seq experiments. CHEs have similar morphology to ionocytes, with long protrusions, and we hypothesize that they are less robust in surviving cell isolation protocols. We have included this in the manuscript in paragraph 2 of the results text.

The data presented was indeed filtered for Epcam, but we have included in the supplemental data a UMAP and dotplot of the unfiltered dataset (Fig S1), demonstrating that our epithelial cells were identified by expression of Epcam.

4) Can the authors quantify the number of BEST+ cells from proximal jejunum to ileum/colon?

We have included these data into Fig 1J, as shown below:

NOTE: We have removed unpublished data that had been provided for the referees in confidence.

5) Figure 2 results are very nice, and the only minor suggestion is in Figure 2B to move the letters from on top the clusters like in other figures. Also to capitalize the word sec_prog so it matches the rest. It might also be worth on the top of Figure 2B to label this "reclustered cells" or circle in 2A which cell types were reclustered for 2B.

Thank you for the suggestions. We have revised as suggested.

6) In Figure 3, the expression of Notch 2 in the single cell data is confusing. The immunofluorescence very nicely supports the presence of various Notch2+ Best4+/- and Notch2+ CFTR+/-, but it would be nice to see this in the single cell data. The Notch-on score is presented, but what about showing expression of individual genes in the pathway, overlayed with expression of Meis1, CFTR, and Best4?

In the original manuscript, we included dotplots of individual genes. However, we have now added Diffusion Maps coded for each individual gene, including Notch2, in the Supplemental data (Fig S4), as shown below:

NOTE: We have removed unpublished data that had been provided for the referees in confidence.

7) Figure 4 has a lot of important data, especially the in vivo experiments and the organoid experiments. What is missing from this figure are descriptions of the experiment being done ie. DAPT injection in vivo, isolation of organoids and then adding DAPT in vitro. That would make this figure much easier to understand. Again DAPI is not labeled.

© 2025. Published by The Company of Biologists under the terms of the Creative Commons Attribution License (https://creativecommons.org/licenses/by/4.0/).

We thank the reviewers for the suggestions. We have made a schematic for the treatment schemes (Fig. 4D& Fig.4I), as shown below:

NOTE: We have removed unpublished data that had been provided for the referees in confidence.

8) It could be helpful to explain a bit more to the reader about the purpose of the study and why rats were chosen as a model organism. This is currently scattered through the manuscript, and it could be consolidated.

Thank you for this suggestion. We have now included a more consolidated rationale for the study and why rats were chosen as a model organism in the introduction.

Reviewer 2: SUMMARY OF THE ADVANCE MADE IN THIS PAPER AND ITS POTENTIAL SIGNIFICANCE TO THE FIELD
This manuscript focuses on uncovering the lineage development of Best4+ cells in the rat intestine. The authors identify these cells as CFTR High Expressors (CHEs), and use scRNAseq, immunohistochemistry, and organoid studies to argue that a "second wave of Notch signaling diversifies the intestinal secretory lineages" to form CHEs. As Best4+ cells are a relatively new intestinal cell type this study is of interest to the field as it describes these cells in detail. Because CHE cells are not found in the mouse, the rat is the most experimentally tractable in vivo model to understand the origin and function of these cells. The study is also of interest because the findings differ from the dominant view from human studies that Best4+ cells are absorptive lineage cells rather than secretory lineage cells. However, although interesting and provocative, the current study does not convincingly support the central theme of the manuscript concluding that CHE cells are secretory lineage cells requiring Notch signaling to be formed. Issues include over reliance on scRNAseq diffusion analysis and immunohistochemistry to suggest that CHE cells are part of the secretory lineage. Further, the results of the Notch pathway inhibition studies suggest that these cells might better fit with an absorptive cell lineage assignment. Thus, although an interesting topic with new information about marker expression in these cells, the studies do not definitively resolve developmental mechanisms for intestinal CHE cell differentiation.

We thank the reviewer for the critical comments and important feedback. We clarify above and within the text that CHEs/Best4+ cells have been predicted in both humans and zebrafish to be more strongly associated with the secretory lineage, consistent with our data and analysis. Intriguingly, but outside the scope of this study, Best4+ cells in the human colon are predicted to arise from the absorptive lineage. We have added mechanistic data, as well as data regarding Notch1 expression, which is associated with the stem cells and absorptive cell specification.

SUGGESTIONS TO AUTHORS
1. The assignment of CHE cells to the secretory lineage is largely based on scRNAseq studies. The diffusion analysis, which is a fundamental part of this study, does not convincingly suggest secretory lineage identify because the CHE cells are disconnected from secretory progenitors in the diffusion plots. Further, interpretations are based on very few cells CHE cells in the scRNAseq dataset. Small cell numbers make predicted lineage connections less secure. Nevertheless, whatever the connection, bioinformatics analysis only promotes a hypothesis that would need to be supported by experimental data. The bioinformatics analysis is not in itself conclusive. And, because the findings contrast with current thinking in the field that these cells are in the absorptive lineage, the experimental data to support this claim need to be more definitive.

Thank you for the thoughtful comments. As shown in Fig 1C and 2A, cells on the absorptive lineage form a continuous connected ribbon or trajectory from stem to the most differentiated enterocyte population (E6). In Fig 2A, the secretory progenitor population is connected to the stem population but forms a distinct path downward compared to the absorptive lineage – Diffusion Component 1 appears to distinguish the absorptive lineage while Diffusion component 2 distinguishes the secretory lineage. While we did not identify clear bridges between either the secretory progenitor population or the absorptive lineage population with CHEs, the EECs and Tuft cell lineages are also disconnected from any progenitors, but we know from previous literature that they arise from the

© 2025. Published by The Company of Biologists under the terms of the Creative Commons Attribution License (https://creativecommons.org/licenses/by/4.0/).

secretory lineage. Consistent with our data, scRNA-seq from human small intestine also suggests that Best4+ cells small intestinal cells arise from the secretory lineage.

We fully agree that experimentally testing the lineage relationship of CHEs to stem cells and known secretory precursors is incredibly valuable. However, the resources available to generate tissue and cell type-specific Cre lines in the rat are limited and do not exist for our cells of interest.

2. The Notch inhibition studies are quite interesting. Both in vivo rat studies and in vitro intestinal organoid studies suggested that CHE cell numbers are reduced when Notch is inhibited by treatment with a gamma secretase (GSI) inhibitor. Notch has been well described to regulate absorptive vs. secretory lineage differentiation in the intestine and GSI treatment promotes secretory cell differentiation and inhibits absorptive cell differentiation. Thus, the findings of this study better align with an absorptive lineage assignment, counter to the proposed secretory lineage assignment of the scRNAseq analysis and the overall conclusion of the study. A switch from Notch off to Notch on for a secretory cell would be of great interest. However, further studies are needed to support the hypothesis that there is a switch in Notch signaling after commitment to the secretory lineage to promote CHE cell differentiation via Notch signaling.

We agree that the requirement for Notch from a secretory lineage cell is unique and unprecedented in the intestine. However, previous studies in human intestine also suggest that small intestinal Best4+ cells arise from secretory progenitors (Burclaff et. al, 2022). Additionally, we have characterized the localization of Notch2+/Prox1+ CHE progenitors along the crypt/villus axis. Notably, they first appear at the +5 position from the crypt base. This is intriguing, as the +4 cell is often considered to be the secretory progenitor and strongly express the Notch ligand Dll1. We confirmed the presence of a Dll1+ proliferative progenitor in rat crypts (Fig S3J-K), but technical limitations with antibodies precludes us from costaining for Dll1 and Notch2 to identify whether the CHE progenitors are specified by direct contact with a Dll1-positive +4 cell. As discussed in more detail below (Reviewer 2, point 5), we have also performed staining for Notch1, which was clearly restricted to stem cells and CHEs. At least for Notch1, we identified interesting staining patterns that are consistent with the re-expression of Notch in CHEs. As shown in Fig 3L, cells in the crypt base expressed Notch1. However, Notch2+/Prox1+ CHE progenitors were Notch1-. Interestingly, mature CHEs in the villus expressed Notch1 protein, consistent with the scRNA-seq analysis that demonstrated Notch1 gene expression in CHEs.

3. There are concerns about experimental rigor. Key studies are based on N=2. For example, the DAPT rat treatment studies do not have statistical power to make definitive conclusions about changes in cell numbers because only 2 rats were included in the analysis. Further, the data suggest incomplete Notch inhibition because the goblet cell hyperplasia was quite modest.

We have increased this to n = 3. We agree that we would expect a more robust increase in goblet cell hyperplasia. However, to avoid DMSO toxicity, we had to use a dose that indeed provided incomplete inhibition of Notch signaling. We have demonstrated this through Hes1 staining of DAPT treated animals (below) and found that while there is a decrease in nuclear Hes1 signal, it is not complete, likely leading to the mild increase in goblet cell abundance.

NOTE: We have removed unpublished data that had been provided for the referees in confidence.

4. The histological analysis of marker expression used to map the expression pattern of proposed CHE lineage cells used sections that are poorly oriented. The sections should show complete crypt-villus cross sections to better support the proposed differentiation process. Further, generalized conclusions are made from high-powered images containing few cells. A more quantitative, morphometric approach is needed to solidify the conclusions of the immunostaining to quantify the various cell states along the crypt-villus axis.

Thank you for this important feedback. We optimized the staining for CHE markers in whole mount intestines in order to robustly capture full crypt-villus axes. We have revised Fig 3 with new images (Fig.3E, G) and added quantifications of fluorescence intensity of each marker based on cell position in the crypt (Fig.3F), as shown below:

© 2025. Published by The Company of Biologists under the terms of the Creative Commons Attribution License (https://creativecommons.org/licenses/by/4.0/).

NOTE: We have removed unpublished data that had been provided for the referees in confidence.

5. The Notch 2 staining pattern is somewhat unexpected which questions the effectiveness of the antibody staining. Other studies would predict that Notch2 would be broadly expressed in absorptive lineage TA cells rather than restricted to a subset of secretory cells. Does the frequency and pattern of antibody stain match Notch2 mRNA expression in the RNAseq data set (note Fig3A does not include the absorptive lineage cells)? Moreover, Notch2 receptor expression does not necessarily indicate active Notch signaling. Is nuclear receptor (NICD) staining observed to support active Notch signaling in CHE cells or proposed CHE precursors?

We have used two distinct Notch2 antibodies and found that they have complete overlapping staining patterns, as shown below. A representative CHE has been marked by a white arrowhead.

NOTE: We have removed unpublished data that had been provided for the referees in confidence.

We have now included FeaturePlots of all epithelial cells coded for expression of Notch1, Notch2, and Hes1 (Fig S4G-I and below).

NOTE: We have removed unpublished data that had been provided for the referees in confidence.

Unlike mouse intestine (Riccio et al, 2005), Notch2 does not appear to be strongly expressed within the crypt of rat intestines. Moreover, we have also stained rat intestines for Notch1 and identified a clear signal within cells in the crypt base (Fig 3L and below). Consistent with our scRNA-seq data, CHEs also express Notch1. Intriguingly, Notch1 does not appear to be expressed in CHE precursors (Notch2+/Prox1+/Best4- cells in crypts).

NOTE: We have removed unpublished data that had been provided for the referees in confidence.

While we did not identify a NICD2 antibody that was suitable for immunofluorescence of rat tissues, we examined nuclear Hes1 expression in CHEs and found strong nuclear signal in CHEs (Fig 4A). Moreover, we have now included data demonstrating that CHE precursors also express nuclear Hes1 (Fig 4C and below). Because our data suggest that Notch1 is not expressed in CHEs until later in maturation, we hypothesize that the main driver of Notch signaling and nuclear Hes1 translocation in CHE precursors is indeed Notch2.

NOTE: We have removed unpublished data that had been provided for the referees in confidence.

6. Organoid studies could provide a system to probe the mechanism to support the proposed conclusions. Can the organoid studies be extended to support the proposed Notch off - Notch on mechanism for CHE differentiation?

We aimed to test this by making Notch2 knockdown lines using lentiviral transduction of shRNA constructs. Unfortunately, screening of 4 separate lines revealed that none were effective in decreasing Notch2 protein levels. We have also spent significant efforts trying to mutate Notch2 in rat organoids using CRISPR. Again, unfortunately, in our hands, the indel rate is 0%, despite selection and the confirmation of Cas9 expression via PCR. In light of the finding that CHEs also express Notch1, we anticipate that Notch1 would compensate for Notch2 loss. Loss of Notch1 AND Notch2 is not feasible due to the stem cells' requirement for Notch-based signaling. Therefore, we did not further pursue this line of experiments.

7. The organoid forskolin secretion experiments are interesting, but do not directly link secretion to CHE cells. DAPT compromises organoid growth and changes differentiation of many different cell types, not just CHE cells.

This is a limitation of the pharmacological Notch inhibition. We aimed to minimize gross defects by performing low-dose short-term DAPT treatment (2 d), where we would see a decrease in CHE number but maintain stem cells in this short window of time. Our data and others suggest that CFTR expression is limited to crypt cells and CHEs, and previous organoid studies have demonstrated that forskolin-based intestinal organoid swelling is entirely through CFTR (Boj et al,

© 2025. Published by The Company of Biologists under the terms of the Creative Commons Attribution License (https://creativecommons.org/licenses/by/4.0/).

2017) Therefore, while we cannot rule out confounding factors, these data are consistent with CHEs performing the majority of fluid transport into the lumen upon forskolin stimulation.

8. The Discussion includes several speculative statements that are not supported by the study. Some examples are listed below.
Page 17 states that "Meis1 likely plays a key role in regulating small intestinal CHE fate, potentially as a terminal differentiation factor capable of upregulating the expression of ion channels required for luminal secretion such as CFTR and Best4."
Page 18 "The behavior of Hes1 between stem/progenitor cells and CHE may represent a transition from oscillatory to sustained Hes1 expression during CHE differentiation."
Page 19 "CHEs may similarly coordinate a regionalized pH regulation in response to lumen acidification."

Thank you for the feedback. We have revised the discussion to reflect the reviewer's concerns.

Reviewer 3: This study used single-cell RNA-seq data from rat intestine to investigate CFTR High Expresser cells (CHEs). These cells are also known in the human intestine as Best4+ cells. They are thought to be derived from the secretory lineage, and are of interest because their functions are still not well-characterized.

The authors investigated transcription factors enriched in this lineage and found that unlike the other secretory cell types like Goblet/Paneth cells, Atoh1 was not expressed in CHEs. Thus, the authors argued that Notch signaling is required in the differentiation of CHEs from the secretory progenitor.
1. The authors asserted that Notch2 is a candidate marker of CHE precursors (Page 10). CHEs are also the only cells expressing high Notch2 (also Page 10). Furthermore, the authors concluded that Notch must be reactivated to specify CHE cells (Page 15). This is an interesting idea that could be explored further by using Notch2 knock-out organoids to see whether they contain CHE cells. This would be a relatively straightforward experiment that can show whether Notch2 is necessary for CHE differentiation.

We agree that in principle, this is a straightforward experiment. However, technically speaking, it has not been. As discussed above (Reviewer 2, point 6), our extensive efforts to knock down or knockout Notch2 in rat intestinal organoids has not been successful. Due to growing concerns that Notch1 would compensate in the absence of Notch2, we have not further pursued these functional tests.

2. The authors performed Forskolin-mediated organoid swelling assay on DAPT-treated organoid, showing that organoids treated with DAPT underwent less swelling (Figure 4K-M). The authors then argued that the difference in swelling can be explained by the lack of CHE cells within organoids treated with DAPT (Page 14). However, considering that even in the authors' data CHE cells only comprise about 1-2% of the cells within the organoid (Figure 4F, J), it is difficult to believe that this change is seen due to the change in composition of the CHE cells.

Considering that enterocytes also express CFTR (as seen in public scRNA-seq data), and that enterocytes are the most abundant in the intestine, what is more likely is that the addition of DAPT reduced the number of enterocytes within the organoids, which in turn affect the swelling of the organoids. Ultimately, the authors did not show in a direct manner that CHE cells were the cause of swelling abrogation.

We thank the reviewer for this valuable feedback and agree it is indeed surprising that comparatively rare CHEs are capable of major effects on organoid swelling. While we cannot rule out a reduction of enterocytes following DAPT addition, Wang et. al (2025) recently demonstrated in human intestinal organoids that swelling induced by the STa analog linaclotide is absent upon complete loss of CHEs induced by genetic ablation of the transcription factor SPIB. This suggests that at least *in vitro*, CHEs may be a major source of fluid secretion, although secretory function for CHEs *in vivo* has yet to be confirmed. Our transcriptomic analysis suggests that SPIB is highly enriched in both CHE and Tuft cell populations (Fig. S3L), supporting the common CHE/Tuft fate trajectory we observed.

© 2025. Published by The Company of Biologists under the terms of the Creative Commons Attribution License (https://creativecommons.org/licenses/by/4.0/).

Second decision letter

MS ID#: dev.204277R1

MS TITLE: Evidence of secondary Notch signaling within the rat small intestine

AUTHORS: Eleanor Zagoren, Nicolas Dias, Anderson K. Santos, Zachary D. Smith, Nadia A. Ameen and Kaelyn Sumigray

Dear Dr Sumigray,

I have now received all the referees reports on the above manuscript, and have reached a decision. The referees' comments are appended below, or you can access them online: please go to.

The overall evaluation is positive and we would like to publish a revised manuscript in Development. However, reviewer 2 had some suggested edits that would help clarify your interpretation of the data and we would like you to address these minor suggestions and submit a revised manuscript at your earliest convenience. Otherwise we are happy with your revisions.

Reviewer 1

The authors have addressed my previous critique well and I feel the paper is suitable for publication.

Reviewer 2

Advance summary and potential significance to field

This study describes a potential developmental lineage for CFTR High Expressor (CHE) cells in the rat intestinal epithelium. They used scRNA-seq to define unique features of these cells and with computational tools proposed a potential lineage origin from secretory progenitors rather than absorptive progenitors. They further demonstrated that development of these cells depends on Notch signaling using functional Notch inhibition studies in rat and rat organoids. The proposed lineage from a Notch-off secretory cell progenitor to a Notch-on developmental lineage is novel. Notably, the analysis of CHE cell development in rat small intestine aligns with previously published scRNA-seq trajectory analysis in human and zebrafish small intestine, although other published studies in human colon have suggested that these cells arise from absorptive progenitors. Thus the developmental lineage of these cells is still unclear (it seems unlikely to me that cell lineage would differ dramatically in small intestine and colon and thus a unifying theory is yet to be determined). Overall the work is well done and nicely described, and the authors were reasonably responsive to the review comments. However, although the data is suggestive of a Notch-off progenitor activating Notch to form CHE cells, the computational scRNA-seq data are not definitive and the discussion should more explicitly state that they can not rule out that these cells may emerge from an absorptive, Notch-on progenitor.

Comments for the author

1. Add a qualifying remark to the Discussion to more explicitly state that CHE cells could emerge from an absorptive, Notch-on progenitor, and that the functional Notch inhibition studies using DAPT would be consistent with this hypothesis.

2. The discussion of Hes1 oscillations at the top of page 19 ("it is possible that a transition from oscillatory Hes1 in progenitors to more sustained Hes1 expression in CHEs could occur") does not really fit with the proposed model of an origin for CHEs from secretory progenitors. Secretory

© 2025. Published by The Company of Biologists under the terms of the Creative Commons Attribution License (https://creativecommons.org/licenses/by/4.0/).

progenitors would be Notch-off and not oscillating. This section should be modified to better align with the proposed mechanism.

Reviewer 3

Advance summary and potential significance to field

1.Although the scRNAseq data is interesting, without experimental data from organoid, it is difficult to support the conclusions of this study regarding the switch in Notch signaling.

2. The paper by Wang et. al (2025) indeed showed abrogation of organoid swelling through knockout of SPIB. This is straightforward, since knockout of SPIB does not change the number of enterocytes within the organoids. However, DAPT is known to reduce the number of enterocytes (which express CFTR) in organoids, therefore, the data shown in this study still do not imply direct causation between the absence of CHE and abrogation of swelling and should be noted as such.

Second revision

Author response to reviewers' comments

We thank the reviewers for their feedback and continued interest in this study. As noted by Reviewers 2 and 3, there are caveats to our study. Per their suggestions, we have revised the text of the manuscript to make these clearer. Point-by-point responses are below.

Reviewer 1: The authors have addressed my previous critique well and I feel the paper is suitable for publication.

We thank the reviewer for their feedback.

Reviewer 2: SUMMARY OF THE ADVANCE MADE IN THIS PAPER AND ITS POTENTIAL SIGNIFICANCE TO THE FIELD

This study describes a potential developmental lineage for CFTR High Expressor (CHE) cells in the rat intestinal epithelium. They used scRNA-seq to define unique features of these cells and with computational tools proposed a potential lineage origin from secretory progenitors rather than absorptive progenitors. They further demonstrated that development of these cells depends on Notch signaling using functional Notch inhibition studies in rat and rat organoids. The proposed lineage from a Notch-off secretory cell progenitor to a Notch-on developmental lineage is novel. Notably, the analysis of CHE cell development in rat small intestine aligns with previously published scRNA-seq trajectory analysis in human and zebrafish small intestine, although other published studies in human colon have suggested that these cells arise from absorptive progenitors. Thus the developmental lineage of these cells is still unclear (it seems unlikely to me that cell lineage would differ dramatically in small intestine and colon and thus a unifying theory is yet to be determined). Overall the work is well done and nicely described, and the authors were reasonably responsive to the review comments. However, although the data is suggestive of a Notch-off progenitor activating Notch to form CHE cells, the computational scRNA-seq data are not definitive and the discussion should more explicitly state that they can not rule out that these cells may emerge from an absorptive, Notch-on progenitor.

SUGGESTIONS TO AUTHORS

1. Add a qualifying remark to the Discussion to more explicitly state that CHE cells could emerge from an absorptive, Notch-on progenitor, and that the functional Notch inhibition studies using DAPT would be consistent with this hypothesis.

© 2025. Published by The Company of Biologists under the terms of the Creative Commons Attribution License (https://creativecommons.org/licenses/by/4.0/).

Thank you for the feedback. We have added the following sentence to the Discussion to qualify that we cannot definitively rule out the absorptive progenitor hypothesis:

“As rats and human models lack similar lineage tracing capabilities, we cannot definitively rule out the possibility that CHEs could arise from an absorptive, "Notch on" progenitor. Indeed, the loss of CHEs upon Notch inhibition we observed would also be consistent with this alternative hypothesis.”

2. The discussion of Hes1 oscillations at the top of page 19 ("it is possible that a transition from oscillatory Hes1 in progenitors to more sustained Hes1 expression in CHEs could occur") does not really fit with the proposed model of an origin for CHEs from secretory progenitors. Secretory progenitors would be Notch-off and not oscillating. This section should be modified to better align with the proposed mechanism.
We thank the reviewer for their critical feedback. We have revised this section to reflect the transition through a Notch-off progenitor, as follows:

“Hes1 is notable for its oscillatory activity (Hirata et al., 2002), which regulates differentiation timing in a variety of systems (Seymour et al., 2020, Maeda et al., 2023). Since Hes1 oscillations have been described in intestinal stem cells (Weterings et. al., 2024), it is possible that CHE specification may involve a transition from oscillatory Hes1 in stem cells, through a Notch off secretory progenitor, and finally towards more sustained Hes1 expression in differentiated CHEs, though this remains to be explored.”

Reviewer 3: 1.Although the scRNAseq data is interesting, without experimental data from organoid, it is difficult to support the conclusions of this study regarding the switch in Notch signaling.

We thank Reviewer 3 for their feedback. We agree that it is essential to genetically manipulate the Notch pathway in organoids to definitively demonstrate the switch in Notch signaling. However, as mentioned, the experiments were technically unachievable for us, and with the Notch1 expression also in CHEs, we hypothesize that there will be compensation in Notch2 KD organoids. Complete loss of Notch results in loss of stem cells, and thus, does not allow for testing our hypothesis. We have modified the discussion throughout the paper to deemphasize the switch in Notch signaling and now add in a statement that we cannot rule out that CHEs arise from the absorptive progenitor (see above).

2. The paper by Wang et. al (2025) indeed showed abrogation of organoid swelling through knockout of SPIB. This is straightforward, since knockout of SPIB does not change the number of enterocytes within the organoids. However, DAPT is known to reduce the number of enterocytes (which express CFTR) in organoids, therefore, the data shown in this study still do not imply direct causation between the absence of CHE and abrogation of swelling and should be noted as such.

Thank you for this constructive feedback. We have revised the text in the discussion of the organoid swelling assays to incorporate that our studies do not imply a direct causation, as follows:

" We observed that DAPT-treated organoids, which exhibit decreased CHE specification, displayed a strong reduction of forskolin induced swelling. However, DAPT is also known to reduce enterocytes, which also express CFTR, and therefore our studies cannot imply a direct causation between the absence of CHEs and the abrogation of swelling."

Third decision letter

MS ID#: dev.204277R2

MS TITLE: Evidence of secondary Notch signaling within the rat small intestine

© 2025. Published by The Company of Biologists under the terms of the Creative Commons Attribution License (https://creativecommons.org/licenses/by/4.0/).

AUTHORS: Eleanor Zagoren, Nicolas Dias, Anderson K. Santos, Zachary D. Smith, Nadia A. Ameen and Kaelyn Sumigray

Dear Dr Sumigray,

I am happy to tell you that your manuscript has been accepted for publication in Development, pending our standard publication integrity checks.

© 2025. Published by The Company of Biologists under the terms of the Creative Commons Attribution License (https://creativecommons.org/licenses/by/4.0/).
